# Supplementary material for: Differences in morphology, mitochondrial genomes, and reproductive compatibility between two clades of parasitic wasps Aphelinus mali (Hymenoptera: Aphelindae) in China
Source: PLoS One. 2023 Feb 2;18(2):e0279663. doi: 10.1371/journal.pone.0279663 (PMC9894431; doi:10.1371/journal.pone.0279663)
Supplement: S1 Fig — (DOCX) [file pone.0279663.s001.docx]

**S1 Fig.** The linear arrangement of mitochondrial genomes in the sequences of two clades


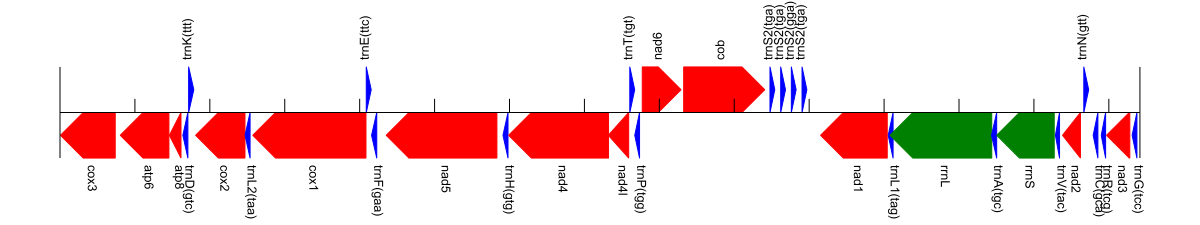


Shandong clade


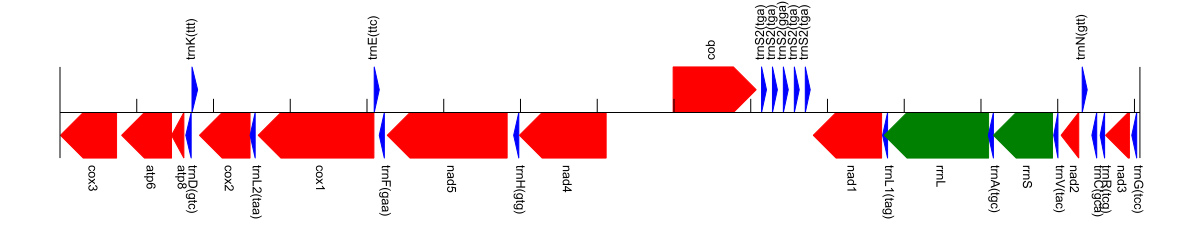


Liaoning clade
