# Supplementary material for: Differences in morphology, mitochondrial genomes, and reproductive compatibility between two clades of parasitic wasps Aphelinus mali (Hymenoptera: Aphelindae) in China
Source: PLoS One. 2023 Feb 2;18(2):e0279663. doi: 10.1371/journal.pone.0279663 (PMC9894431; doi:10.1371/journal.pone.0279663)
Supplement: S1 Table — (DOCX) [file pone.0279663.s002.docx]

S1 Table. Primers used for amplification of the mitochondrial genome

| **Fragment**  **No.** | **Gene or**  **region** | **Primer**  **name** | **Sequence (5’-3’)** | **Length(bp)** |
| --- | --- | --- | --- | --- |
| F1 | *tRNA-Cys-COX3* | RGF1 | CCAAWTAAGAAATATAAATATTC | 871 |
|  |  | RGR1 | CTGGTTTTCATGGTCTTCATG |  |
| F2 | *COX3* | RGF2 | GTCAATATCAAGCTGCAGC | 191 |
|  |  | RGR2 | AGAGTATTCTGAAGCTTC |  |
| F3 | *COX3-ATP6* | RGF3 | GTTCCTATTAAAACATGAAGACC | 926 |
|  |  | RGR3 | CCTTTAGGGACACCAGGAG |  |
| F4 | *ATP6* | RGF4 | GACCAGCAATCATATTTGCAG | 486 |
|  |  | RGR4 | CTGTTTTTGATCCTTCTAC |  |
| F5 | *ATP6-COX1* | RGF5 | ATAACGAGAAGGAATAAATC | 1876 |
|  |  | RGR5 | GAATGGATGTAGATACTCG |  |
| F6 | *COX1* | RGF6 | CCATTTATTGAAGCAAGTCATC | 616 |
|  |  | RGR6 | CTGGGACAGGTTGAACTG |  |
| F7 | *COX1-NAD5* | RGF7 | TTGAACCTATAATTGAAG | 1346 |
|  |  | RGR7 | GTCAAGATATTCGTTTTATAGG |  |
| F8 | *NAD5* | RGF8 | AAGGTATTCCACATAATG | 710 |
|  |  | RGR8 | TGGGTGGGATGGTTTAGG |  |
| F9 | *NAD5-NAD4* | RGF9 | TATTACATCTCCAATTCG | 1073 |
|  |  | RGR9 | GTTTGTTTACGTCAGTATG |  |
| F10 | *NAD4* | RGF10 | GCTGAAGAACATAAACCATG | 331 |
|  |  | RGR10 | GACTTCCTAAAGCTCATG |  |
| F11 | *NAD4-CYTB* | RGF11 | CGAACAACACCATAACCC | 2239 |
|  |  | RGR11 | GAATAAAATCGATTTAGTGTAGC |  |
| F12 | *CYTB* | RGF12 | TGAGGAGCTACTGTTATTAC | 396 |
|  |  | RGR12 | TCATTCAGGTTGAATATG |  |
| F13 | *CYTB-NAD1* | RGF13 | CCATATATTTTAAGAGACCC | 1475 |
|  |  | RGR13 | GATTTATAATAATTAGGTTTCCAG |  |
| F14 | *NAD1* | RGF14 | CCTTTCATATATTCTGTG | 397 |
|  |  | RGR14 | GAAATAATTTACCATTTTATTCTC |  |
| F15 | *NAD1-RRNL* | RGF15 | GAGCAATACTCCGAAGAC | 697 |
|  |  | RGR15 | GTTTGCGACCTCGATGTTG |  |
| F16 | *RRNL* | RGF16 | CTCACGCCGATCTAAACTC | 1051 |
|  |  | RGR16 | CCTTTTGTATCAGGGTTA |  |
| F17 | *RRNL-RRNS* | RGF17 | TTAATCATTGAGCAGATC | 871 |
|  |  | RGR17 | GTACAAATTGCCCGTCGC |  |
| F18 | *RRNS* | RGF18 | ACTATATTCCTAACTTCT | 461 |
|  |  | RGR18 | AACTAGGATTAGATACC |  |
| F19 | *RRNS-tRNA-Asn* | RGF19 | CAATTACAAAACAAGTTCCTC | 904 |
|  |  | RGR19 | CTTAATTGGAAAAATAATTTC |  |
