# Supplementary material for: Differences in morphology, mitochondrial genomes, and reproductive compatibility between two clades of parasitic wasps Aphelinus mali (Hymenoptera: Aphelindae) in China
Source: PLoS One. 2023 Feb 2;18(2):e0279663. doi: 10.1371/journal.pone.0279663 (PMC9894431; doi:10.1371/journal.pone.0279663)
Supplement: S2 Table — (DOCX) [file pone.0279663.s003.docx]

S2 Table. Comparisons of the mitochondrial genome between two clades of *Aphelinus mali* in China

| Gene | Clade | Strand | Length(bp） | Anti/start codon | Stop codon | Intergenic nucleotides |
| --- | --- | --- | --- | --- | --- | --- |
| *COX3* | Shandong | - | 732 | ATG | TAA | 39 |
|  | Liaoning | - | 732 | ATG | TAA | 2 |
| *ATP6* | Shandong | - | 651 | ATT | TAA | 3 |
|  | Liaoning | - | 651 | ATT | TAG | -10 |
| *ATP8* | Shandong | - | 156 | ATT | TAA | -10 |
|  | Liaoning | - | 162 | ATT | TAA | 24 |
| *TRND* | Shandong | - | 68 | GTC |  | 24 |
|  | Liaoning | - | 71 | GTC |  | 10 |
| *TRNK* | Shandong | + | 70 | TTT |  | 9 |
|  | Liaoning | + | 74 | TTT |  | 2 |
| *COX2* | Shandong | - | 660 | ATT | TAA | 2 |
|  | Liaoning | - | 660 | ATT | TAA | 0 |
| *TRNL2* | Shandong | - | 68 | TAA |  | 0 |
|  | Liaoning | - | 67 | TAA |  | 14 |
| *COX1* | Shandong | - | 1512 | ATT | TAA | -18 |
|  | Liaoning | - | 1509 | ATT | TAA | -18 |
| *TRNE* | Shandong | + | 67 | TTC |  | 4 |
|  | Liaoning | + | 67 | TTC |  | -1 |
| *TRNF* | Shandong | - | 70 | GAA |  | -1 |
|  | Liaoning | - | 67 | GAA |  | 0 |
| *NAD5* | Shandong | - | 1479 | ATT | TAA | 11 |
|  | Liaoning | - | 1563 | ATT | T | 12 |
| *TRNH* | Shandong | - | 68 | GTG |  | 0 |
|  | Liaoning | - | 68 | GTG |  | 0 |
| *NAD4* | Shandong | - | 1335 | ATG | T | -7 |
|  | Liaoning | - | 1308 | ATG | T | -7 |
| *NAD4L* | Shandong | - | 273 | ATA | TAA | -7 |
|  | Liaoning | - | 267 | ATA | TAA | -7 |
| *TRNT* | Shandong | + | 66 | TGT |  | 0 |
|  | Liaoning | + | 66 | TGT |  |  |
| *TRNP* | Shandong | - | 65 | TGG |  | 2 |
|  | Liaoning | - | 64 | TGG |  |  |
| *NAD6* | Shandong | + | 519 | ATA | T | 15 |
|  | Liaoning | + | 513 | ATA | T | 1 |
| Gene | Clade | Strand | Length(bp） | Anti/start codon | Stop codon | Intergenic nucleotides |
| *COB* | Shandong | + | 1083 | ATA | TAA | 1 |
|  | Liaoning | + | 1083 | ATA | TAA | 27 |
| *TRNS2* | Shandong | + | 65 | TGA |  | 27 |
|  | Liaoning | + | 65 | TGA |  | 73 |
| *TRNS2* | Shandong | + | 65 | TGA |  | 77 |
|  | Liaoning | + | 70 | TGA |  | 77 |
| *TRNS2* | Shandong | + | 65 | GGA |  | 78 |
|  | Liaoning | + | 65 | GGA |  | 77 |
| *TRNS2* | Shandong | + | 65 | TGA |  | 149 |
|  | Liaoning | + | 65 | TGA |  | 149 |
| *NAD1* | Shandong | - | 891 | ATA | TAA | 0 |
|  | Liaoning | - | 891 | ATA | TAA | 0 |
| *TRNL1* | Shandong | - | 66 | TAG |  | 0 |
|  | Liaoning | - | 66 | TAG |  | 0 |
| *RRNL* | Shandong | - | 1359 |  |  | 0 |
|  | Liaoning | - | 1360 |  |  | 0 |
| *TRNA* | Shandong | - | 70 | TGC |  | 0 |
|  | Liaoning | - | 67 | TGC |  | 0 |
| *RRNS* | Shandong | - | 769 |  |  | 0 |
|  | Liaoning | - | 771 |  |  | 0 |
| *TRNV* | Shandong | - | 56 | TAC |  | 323 |
|  | Liaoning | - | 56 | TAC |  | 20 |
| *NAD2(P)* | Shandong | - | 240 | ATT | ATT | 40 |
|  | Liaoning | - | 213 | ATT | TGA |  |
| *TRNN* | Shandong | + | 66 | GTT |  | 0 |
|  | Liaoning | + | 66 | GTT |  |  |
| *TRNC* | Shandong | - | 64 | GCA |  |  |
|  | Liaoning | - | 64 | GCA |  | 44 |
| *TRNR* | Shandong | - | 59 | TCG |  |  |
|  | Liaoning | - | 59 | TCG |  | 2 |
| *NAD3* | Shandong | - | 312 | ATA | TAA |  |
|  | Liaoning | - | 312 | ATA | TAA | -3 |
| *TRNG* | Shandong | - | 65 | TCC |  |  |
|  | Liaoning | - | 65 | TCC |  | 41 |
| // | Shandong |  | 998 |  |  |  |
|  | Liaoning |  | 972 |  |  |  |
