# Supplementary material for: Differences in morphology, mitochondrial genomes, and reproductive compatibility between two clades of parasitic wasps Aphelinus mali (Hymenoptera: Aphelindae) in China
Source: PLoS One. 2023 Feb 2;18(2):e0279663. doi: 10.1371/journal.pone.0279663 (PMC9894431; doi:10.1371/journal.pone.0279663)
Supplement: S3 Table — (DOCX) [file pone.0279663.s004.docx]

S3 Table. The base content, AT skewness and GC skewness of the two *Aphelinus mali* clades compared to other Hymenoptera

| Species | T% | C% | A% | G% | A+T% | ATskew | GCskew |
| --- | --- | --- | --- | --- | --- | --- | --- |
| *Aphelinus mali SC* | 36.69 | 9.40 | 47.30 | 6.61 | 84.44 | 0.1291 | -0.1619 |
| *Aphelinus mali LC* | 36.69 | 9.40 | 47.30 | 6.61 | 83.99 | 0.1305 | -0.1328 |
| *Taeniogonalos taihorina* | 43.51 | 6.77 | 41.09 | 8.63 | 84.60 | -0.0286 | 0.1208 |
| *Spathius agrili* | 45.12 | 6.55 | 38.88 | 9.45 | 84.00 | -0.0743 | 0.1813 |
| *Cotesia vestalis* | 47.46 | 5.78 | 39.70 | 7.06 | 87.16 | -0.0890 | 0.0997 |
| *Enicospilus* sp. | 43.44 | 8.73 | 41.45 | 6.08 | 85.19 | -0.0198 | -0.1789 |
| *Abispa ephippium* | 39.50 | 14.60 | 39.10 | 6.70 | 78.60 | -0.0051 | -0.3709 |
| *Bombus hypocrita* | 42.56 | 9.68 | 42.77 | 4.98 | 85.33 | 0.0025 | -0.0275 |
| *Perga condei* | 33.80 | 14.60 | 42.80 | 8.80 | 76.60 | 0.1175 | -0.2479 |

SC: Shandong clade, LC: Liaoning clade
