# Supplementary material for: Differences in morphology, mitochondrial genomes, and reproductive compatibility between two clades of parasitic wasps Aphelinus mali (Hymenoptera: Aphelindae) in China
Source: PLoS One. 2023 Feb 2;18(2):e0279663. doi: 10.1371/journal.pone.0279663 (PMC9894431; doi:10.1371/journal.pone.0279663)
Supplement: S4 Table — (DOCX) [file pone.0279663.s005.docx]

S4 Table. The base composition of mitochondrial genomic coding gene and rRNA gene of two clades of *Aphelinus mali* in China

| Gene | Clades | T％ | C％ | A％ | G％ | A＋T％ | AT skew | GC skew |
| --- | --- | --- | --- | --- | --- | --- | --- | --- |
| *COX3* | Shandong | 30.13 | 12.05 | 49.23 | 8.59 | 79.36 | 0.2454 | -0.1676 |
|  | Liaoning | 29.87 | 12.44 | 49.10 | 8.59 | 78.97 | 0.2435 | -0.1831 |
| *ATP6* | Shandong | 30.83 | 8.85 | 50.88 | 9.44 | 81.71 | 0.2454 | -0.0323 |
|  | Liaoning | 31.56 | 9.29 | 50.15 | 9.00 | 81.71 | 0.2282 | -0.0159 |
| *ATP8* | Shandong | 42.42 | 4.85 | 49.09 | 3.64 | 91.52 | 0.0729 | -0.1427 |
|  | Liaoning | 41.82 | 4.85 | 49.09 | 4.24 | 90.91 | 0.0800 | -0.0671 |
| *COX2* | Shandong | 34.51 | 11.01 | 46.26 | 8.22 | 80.76 | 0.1455 | -0.1450 |
|  | Liaoning | 34.80 | 11.01 | 45.81 | 8.37 | 80.62 | 0.1366 | -0.1362 |
| *COX1* | Shandong | 31.66 | 12.81 | 45.75 | 9.78 | 77.41 | 0.1820 | -0.1341 |
|  | Liaoning | 30.82 | 13.32 | 46.27 | 9.59 | 77.09 | 0.2004 | -0.1628 |
| *NAD5* | Shandong | 33.21 | .72 | 52.21 | 5.85 | 85.42 | 0.2224 | -0.1968 |
|  | Liaoning | 33.13 | 8.49 | 52.63 | 5.74 | 85.77 | 0.2274 | -0.1933 |
| *NAD4* | Shandong | 34.28 | 8.79 | 50.67 | 6.26 | 84.95 | 0.1929 | -0.1681 |
|  | Liaoning | 32.11 | 11.60 | 47.01 | 9.28 | 79.12 | 0.1883 | -0.1111 |
| *NAD4L* | Shandong | 34.01 | 8.42 | 54.55 | 3.03 | 88.55 | 0.2320 | -0.4707 |
|  | Liaoning | 35.35 | 7.74 | 54.88 | 2.02 | 90.23 | 0.2164 | -0.5860 |
| *NAD6* | Shandong | 45.82 | 6.00 | 44.91 | 3.27 | 90.73 | -0.0100 | -0.2945 |
|  | Liaoning | 44.91 | 6.79 | 45.06 | 3.24 | 89.97 | 0.0017 | -0.3539 |
| *COB* | Shandong | 43.73 | 10.93 | 35.29 | 10.04 | 79.02 | -0.1068 | -0.0424 |
|  | Liaoning | 42.81 | 11.58 | 35.53 | 10.09 | 78.33 | -0.0929 | -0.0688 |
| *NAD1* | Shandong | 33.76 | 11.75 | 47.44 | 7.05 | 81.20 | 0.1685 | -0.2500 |
|  | Liaoning | 33.44 | 12.39 | 47.22 | 6.94 | 80.66 | 0.1708 | -0.2818 |
| *NAD3* | Shandong | 33.33 | 9.32 | 52.26 | 5.08 | 85.59 | 0.2212 | -0.2942 |
|  | Liaoning | 33.33 | 9.60 | 51.41 | 5.65 | 84.75 | 0.1661 | -0.2590 |
| *RRNL* | Shandong | 41.20 | 7.46 | 46.76 | 4.57 | 87.97 | 0.0632 | -0.2352 |
|  | Liaoning | 41.08 | 7.55 | 46.95 | 4.42 | 88.03 | 0.0667 | -0.2615 |
| *RRNS* | Shandong | 42.39 | 7.67 | 46.42 | 3.51 | 88.82 | 0.0454 | -0.3721 |
|  | Liaoning | 42.15 | 7.65 | 46.82 | 3.37 | 88.98 | 0.0525 | -0.3884 |
| *NAD2* | Shandong | 35.00 | 5.00 | 55.00 | 5.00 | 90.00 | 0.2222 | 0 |
|  | Liaoning | 35.92 | 4.92 | 54.93 | 4.23 | 90.85 | 0.2092 | -0.0754 |
