# Supplementary material for: Differences in morphology, mitochondrial genomes, and reproductive compatibility between two clades of parasitic wasps Aphelinus mali (Hymenoptera: Aphelindae) in China
Source: PLoS One. 2023 Feb 2;18(2):e0279663. doi: 10.1371/journal.pone.0279663 (PMC9894431; doi:10.1371/journal.pone.0279663)
Supplement: Attachment — Submitted filename: PONE-D-21-21080_reviewer comments (due Sept 27)_9-23-21.pdf [file pone.0279663.s006.pdf]

PLOS ONE

Differences in morphology, mitochondrial genomes, and reproductive compatibility between two clades of Aphelinus mali (Hemiptera: Aphididae) in China

--Manuscript Draft--

**Manuscript Number:**	PONE-D-21-21080
**Article Type:**	Research Article
**Full Title:**	Differences in morphology, mitochondrial genomes, and reproductive compatibility between two clades of Aphelinus mali (Hemiptera: Aphididae) in China
**Short Title:**	Differences between two clades of Aphelinus mali (Hemiptera: Aphididae) in China
**Corresponding Author:**	Hongxu Zhou<br>Qingdao Agricultural University<br>Qingdao, Shandong CHINA
**Keywords:**	Aphelinus mali, hybridization, morphological characteristics, mitochondrial genome
**Abstract:**	Aphelinus mali (Haldeman) (Hymenoptera: Aphelinidae) in China is comprised of two clades (termed, the Shandong and Liaoning clades). In order to clarify the genetic relationship between them, we compared and analyzed the morphological characteristics and the mitochondrial genome of each, and ran a hybridization experiment between these clades . Morphological results showed that both males and females of the Liaoning clade were larger than in the Shandong clade, in terms of whole body, abdominal, wing and antennal lengths, but there were no significant differences between clades for total length of the middle or hind of females. The length of the mitochondrial genome of the Shandong clade was14415 bp and, for the Liaoning clade, it was 14804 bp. Each contained 31 genes, including 13 protein-encoded genes, 16 tRNA genes, and 2 rRNA genes. The highest ATlevels among the 13 protein-coding genes for the two clades were for the same gene (atp8) (Shandong clade, 91.5%; Liaoning clade, 90.9%). By hybridization and backcrossing, we found there was no cross incompatibility between these two clades of A. mali . Our results indicate that the historic geographical isolation between these clades has not yet caused reproductive isolation of these populations, and they belong to the same species.
**Order of Authors:**	Min Su
	Lingjun Du
	Yasir Ali Muhammad
	Jianing Yu
	Mengyu Chi
	Ziwen Teng
	Yinjun Fan
	Xiumei Tan
	Hongxu Zhou
**Additional Information:**	
**Question**	**Response**
**Financial Disclosure**<br><br>Enter a financial disclosure statement that describes the sources of funding for the work included in this submission. Review the submission guidelines for detailed requirements. View published research articles from *PLOS ONE* for specific	National Natural Science Foundation (31772232) .<br>Taishan Mountain Scholar Constructive Engineering Foundation of Shandong, China

Powered by Editorial Manager® and ProduXion Manager® from Aries Systems Corporation

<table>
<tr><td>examples.

This statement is required for submission and will appear in the published article if the submission is accepted. Please make sure it is accurate.

Unfunded studies
Enter: The author(s) received no specific funding for this work.

Funded studies
Enter a statement with the following details:
• Initials of the authors who received each award
• Grant numbers awarded to each author
• The full name of each funder
• URL of each funder website
• Did the sponsors or funders play any role in the study design, data collection and analysis, decision to publish, or preparation of the manuscript?
• NO - Include this sentence at the end of your statement: The funders had no role in study design, data collection and analysis, decision to publish, or preparation of the manuscript.
• YES - Specify the role(s) played.

* typeset</td><td></td></tr>
<tr><td>Competing Interests

Use the instructions below to enter a competing interest statement for this submission. On behalf of all authors, disclose any competing interests that could be perceived to bias this work—acknowledging all financial support and any other relevant financial or non-financial competing interests.

This statement is required for submission and will appear in the published article if the submission is accepted. Please make sure it is accurate and that any funding sources listed in your Funding Information later in the submission form are also declared in your Financial Disclosure statement.</td><td>The authors have declared that no competing interests exist.</td></tr>
</table>

View published research articles from *PLOS ONE* for specific examples.<br><br>**NO authors have competing interests**<br><br>Enter: *The authors have declared that no competing interests exist.*<br><br>**Authors with competing interests**<br><br>Enter competing interest details beginning with this statement:<br><br>*I have read the journal's policy and the authors of this manuscript have the following competing interests: [insert competing interests here]*<br><br>* typeset	
**Ethics Statement**<br><br>Enter an ethics statement for this submission. This statement is required if the study involved:<br><br>• Human participants<br>• Human specimens or tissue<br>• Vertebrate animals or cephalopods<br>• Vertebrate embryos or tissues<br>• Field research<br><br>Write "N/A" if the submission does not require an ethics statement.<br><br>General guidance is provided below. Consult the submission guidelines for detailed instructions. **Make sure that all information entered here is included in the Methods section of the manuscript.**	N/A

**Format for specific study types**<br><br>**Human Subject Research (involving human participants and/or tissue)**<br>• Give the name of the institutional review board or ethics committee that approved the study<br>• Include the approval number and/or a statement indicating approval of this research<br>• Indicate the form of consent obtained (written/oral) or the reason that consent was not obtained (e.g. the data were analyzed anonymously)<br><br>**Animal Research (involving vertebrate animals, embryos or tissues)**<br>• Provide the name of the Institutional Animal Care and Use Committee (IACUC) or other relevant ethics board that reviewed the study protocol, and indicate whether they approved this research or granted a formal waiver of ethical approval<br>• Include an approval number if one was obtained<br>• If the study involved *non-human primates*, add *additional details* about animal welfare and steps taken to ameliorate suffering<br>• If anesthesia, euthanasia, or any kind of animal sacrifice is part of the study, include briefly which substances and/or methods were applied<br><br>**Field Research**<br><br>Include the following details if this study involves the collection of plant, animal, or other materials from a natural setting:<br>• Field permit number<br>• Name of the institution or relevant body that granted permission	
**Data Availability**<br><br>Authors are required to make all data underlying the findings described fully available, without restriction, and from the time of publication. PLOS allows rare exceptions to address legal and ethical concerns. See the PLOS Data Policy and FAQ for detailed information.	Yes - all data are fully available without restriction

A Data Availability Statement describing where the data can be found is required at submission. Your answers to this question constitute the Data Availability Statement and **will be published in the article**, if accepted.<br><br>**Important:** Stating 'data available on request from the author' is not sufficient. If your data are only available upon request, select 'No' for the first question and explain your exceptional situation in the text box.<br><br>Do the authors confirm that all data underlying the findings described in their manuscript are fully available without restriction?	
**Describe where the data may be found in full sentences. If you are copying our sample text, replace any instances of XXX with the appropriate details.**<br><br>• If the data are **held or will be held in a public repository**, include URLs, accession numbers or DOIs. If this information will only be available after acceptance, indicate this by ticking the box below. For example: *All XXX files are available from the XXX database (accession number(s) XXX, XXX.).*<br>• If the data are all contained **within the manuscript and/or Supporting Information files**, enter the following: *All relevant data are within the manuscript and its Supporting Information files.*<br>• If neither of these applies but you are able to provide **details of access elsewhere**, with or without limitations, please do so. For example:<br><br>*Data cannot be shared publicly because of [XXX]. Data are available from the XXX Institutional Data Access / Ethics Committee (contact via XXX) for researchers who meet the criteria for access to confidential data.*<br><br>*The data underlying the results presented in the study are available from (include the name of the third party*	All relevant data are within the manuscript and its Supporting Information files.

and contact information or URL).<br>• This text is appropriate if the data are owned by a third party and authors do not have permission to share the data.<br><br>* typeset	
Additional data availability information:	

Manuscript

Click here to access/download;Manuscript;6.28-Su- edit.docx

Differences in morphology, mitochondrial genomes, and reproductive compatibility between two clades of *Aphelinus mali* (Hemiptera: Aphididae) in China

Su Min[a,b], Du Lingjun[a], Muhammad Yasir Ali[a], Yu Jianing[a], Chi Mengyu[a], Teng Ziwen[a], Fan Yinjun[a], Tan Xiumei[a], Zhou Hongxu[a,*]

[a]College of Botanical Medicine, Qingdao Agricultural University, Key Laboratory of Integrated Plant Disease and Pest Control, China-Australia Joint Institute of Agricultural and Environmental Health, Qingdao 266109, China (18765903010@163.com; 1163355473@qq.com; m.yasirali4663@gmail.com; 2354846762@qq.com; 1538848218@qq.com; tzwbat@126.com; fanyinjun89@126.com; xmtanly@163.com; hxzhouqd@sina.cn)

[b]Agricultural and rural Bureau of Zhangqiu District, Jinan 250200, China (18765903010@163.com)

* Corresponding author(hxzhouqd@sina.cn)

Su Min helped write the article; Du Lingjun contributed the work on the mitochondrial genomes; Yu Jianing contributed to the part on reproductive compatibility; Chi Mengyu contributed to the part on morphological characteristics.

Abstract

Aphelinus mali (Haldeman) (Hymenoptera: Aphelinidae) in China is comprised of two clades (termed, the Shandong and Liaoning clades). In order to clarify the genetic relationship between them, we compared and analyzed the morphological characteristics and the mitochondrial genome of each, and ran a hybridization experiment between these clades. Morphological results showed that both males and females of the Liaoning clade were larger than in the Shandong clade, in terms of whole body, abdominal, wing and antennal lengths, but there were no significant differences between clades for total length of the middle or hind of females. The length of the mitochondrial genome of the Shandong clade was14415 bp and, for the Liaoning clade, it was 14804 bp. Each contained 31 genes, including 13 protein-encoded genes, 16 tRNA genes, and 2 rRNA genes. The highest ATlevels among the 13 protein-coding genes for the two clades were for the same gene (*atp8)* (Shandong clade, 91.5%; Liaoning clade, 90.9%). By hybridization and backcrossing, we found there was no cross incompatibility between these two clades of *A. mali*. Our results indicate that the historic geographical isolation between these clades has not yet caused reproductive isolation of these populations, and they belong to the same species.

Key words *Aphelinus mali*, hybridization, morphological characteristics, mitochondrial genome

Introduction

Woolly apple aphid, *Eriosoma lanigerum*(Hausmann) (Hymenoptera: Aphididae), is an important quarantine pest of apples in many parts of the world (Jaume et al. 2015). In recent years, the damage caused by this aphid has become a serious problem in China, and the pest's range has continued to increase (Zhang and Luo 2002, Chen et al.2006). The endoparasitoid *Aphelinus mali* (Haldeman) (Hymenoptera: Aphelinidae) is the dominant natural enemy of *E. lanigerum* in China (Zhou et al. 20214) and is considered the most effective of the aphid's bio control agents (Yang et al. 2005, Kuang et al. 1985) . *A. mali* has been introduced into 51 countries and has established in 42 of them (Ma et al. 2008).

A. mali was introduced into China twice: the first introduction was from Japan into Dalian and Lvshun, Liaoning Province in 1942, while the second was from the former Soviet Union in 1950 into Qingdao, Shandong Province (Long et al. 1960). High intra-specific variability is expected when a species has a large geographic distribution, as does *A. mali* (Maxwell et al. 2008). Based on the study of the mitochondrial COI gene of this parasitoid, we found that *A. mali* in China is comprised of two regional clades (named the Shandong and Liaoning clades), and that the *A. mali* population in Qingdao and Tai'an in Shandong province and the population in Dalian and Huludao in Liaoning province (Zhang et al. 2014, Zhou et al. 2014) differ in their biological characteristics and control potential (Su et al. 2017, Su et al. 2018).

The study of the genetic relationship between these two clades can help to assess

the ecological adaptability of each clade and determine whether they have undergone intra-specific differentiation. Such information can provide new ideas and methods for the introduction, reproduction, and field release of *A.mali* as a biological control agent against *E. lanigerum*. The morphological characteristics and mitochondrial genome were compared to clarify the genetic relationships between two clades of *A.mali*. We also ran a hybridization experiment to see if any reproduction isolation has developed between these clades.

Materials and Methods

Morphological comparison of clades. To obtain parasitoids for morphological comparisons, we collected *E. lanigerum* aphids from apple orchards in Tai'an, Shandong province and Dalian, Liaoning province, in mid-August, 2014. Parasitized aphids were noted and placed in petri dishes and held atca25°C for parasitoid emergence. Adults of *A. mali* were removed daily , placed in 100% ethanol, and stored at 20°C. Male and female *A. mali* were separated based on the larger body size of females, antennal shape (the third section of the female's antenna at the funicular joint is square, while that of the male is rectangular) (Su et al. 2016), and the abdominal shape (females have a short, thick abdomen, while the male's abdomen is slender) (Long et al. 1960).

Images of body regions or parts were taken with the universal video imaging system(LY-WN-HPCCD (10),Chengdu Li Yang Precision Mechanical and Electrical Co., Ltd., China). This system was used to capture photographs of morphological parts, and we then measured the length of the body and abdomen, the length and

width of front and hind wing, the length of the whole leg, the tibia, tarsae, anterior tarsal segment, and calcar (spine at the tip of the tibia) of the [illegible], middle, and hind legs, the length and width of th[illegible]d, and [illegible]st, the second, and the third antennal segments. One hundred *A. mali* (50M and 50F) from each sample area were measured.

Mitochondrial genomic sequencing. For mitochondrial sequencing, we collected aphids parasitized by *A. mali* from one abandoned apple orchard in Tai'an, Shandong Province and another in Hulud[illegible], Liaoning province. At each site, the five-point sampling method was used, sampling ten trees at each of the five points. On each of these 50 trees per location, five branches bearing blackened *E. lanigerum* aphids that were parasitized by *A. mali* were collected and put into a 25°Cincubator in the laboratory f[illegible]. *mali* emergence. One hundred adults of *A. mali* were obtained from each site and placed individually in 1.5 ml centrifuge tubes containing absolute ethanol. and sent to Wuhan Transduction Bio Co., LTD for sequencing.

For DNA extraction, we used the Takara Genomic extraction Kit, specific Primer LA Taq Polymerase, $MgCl_2$ solution and pMD18-T Vector. For sequencing we used the following procedure. First, primers were designed for the general conserved region to amplify the short regions of the 16s, 12s, cox1, atp6, cox3, nad4, cytb, and nad1 genes, and then specific primers were designed to amplify the remaining sequences according to the amplified conserved sequences. The resulting long fragments were then amplified using LA Taq polymerase, and the PCR program was run through 35 cycles (specific process: 94°C for 30 s, annealed at 50°C for 30s,

extension at 72°C for 1 min per 1kb.). The PCR product was then cloned into a PMD-18T (Takara, JAP) vector or sequenced directly with PCR product in ABI3730 automatic sequencer. The sequencing results were then spliced and assembled by DNASTAR software, and the complete mitochondrial sequence was obtained by manual analysis and adjustment. For amplification of the product, we then added 0.5μLTaKaRa LA Taq (5U/μL), 5μL 10×LA Taq BufferII (Mg^{2+}Plus), 8 μL dNTP Mixture (2.5 mM each), 60 ng Template, 0.2 -1.0μm (final conc.), Primer F and Primer R, 0.2 -1.0μm (final conc.), adding ddH_2O to make up the solution to 50 μL.

Cross mating experiment. For the cross-mating experiment, we collected *A.mali* as parasitized aphids on apple trees (as described below) from Qingdao to represent the Shandong clade and from Qinhuangdao from the Liaoning clade.

We choose an apple orchard with less management and protected the apple trees that had colonies of *E. lanigerum* with screen cages (1m×1m×2m). Thereafter, we p[illegible] out the parasitized black aphids daily until [illegible]oesn't appear, make sure there were no *A. mali* in the *E. lanigerum* colon.

Crosses and reciprocal crosses between the two populations (one per clade) were made using within clade crosses in each population as the control group (Liaoning♀×Shandong♂, Liaoning♂×Shandong♀); all crosses were repeated 15-20 times. The *A. mali* were held in the Petri dishes(13.5 cm diam, had cotton ball with 10% honey water) at 25℃, 70%RH, and a 16:8 L:D photoperiod. For each cross, we recorded the number of hosts attacked by each mated, crossed female and the number of her offspring, and these offspring's sex ratio (as % female).

The F1 generation of each hybrid cross were backcrossed with the parents of each population, conducting a “within hybrid (F1) population” cross as the control group, with 3-5 replicates per type of crossing. We recorded the number of hosts attacked and the number of parasitoid offspring for each crossed female, for all crossing combinations, as well as the sex ratio of the resulting backcrossed progeny (as % female).

In each cross, the numbers of male and female progeny were counted and the mean sex ratio (MSR) for that cross was calculated as the percentage of female progeny. The relative compatibility of an inter-group crossing (A x B) was expressed as (Pinto et al. 1991, Shi et al. 2002):

$$\frac{\mathrm{MSR}(\mathrm{A}♀ \times \mathrm{B}♂)}{\mathrm{MSR}(\mathrm{A}♀ \times \mathrm{A}♂)} \times 100\%$$

Statistical analysis. Morphological measurements as well as the hybridization and backcross rates are presented as means ± standard deviations (SD), calculated using Statistical Product and Service Solutions(SPSS) 19.0.Significant differences were determined using one-way analysis of variance(ANOVA) corrected by SPSS 19.0. The independent samples t-test was used to analyze the data on parthenogenesis between two clades, using SPSS 19.0

For genomic analysis, we used MITOS Webserver (http:// mitos. bioinf. uni-leipzig. de/index. py) to annotate and analyze the mitochondrial genome sequence and obtain the linear alignment of the mitochondrial genome sequence. The AT-skew and GC-skew were calculated using DNASTAR as follows: AT-skew = (A% -T%)/ (A% +T%) and GC-skew = (G%-C %)/ (G%+C%).

Results

Comparison of morphological characters. Measurements of body dimensions (Table 1) showed that both males and females from the Liaoning clade were bigger than those of the Shandong clade, in terms of the whole body, abdominal, wing, and antennal segment lengths. But the total length of the middle and hind legs showed no significant differences in females between two clades.

Mitochondrial genome sequencing and analysis. The mitochondrial genome had 14415 bp for the Shandong clade and 14804 bp for Liaoning. Both clades contained 31 genes, including 13 protein-encoded genes, 16 tRNA genes, and 2 rRNA genes(Table 2, Fig.1). The unmeasured area was in the A+T-rich region, which usually contains six tRNA genes (*trnY*, *trnM*, *trnI*, *trnQ*, *trnW*, and *trnS1*) in such genomes, but which were absent in *A. mali*. Genomes of both clades had a higher A+T content and AT skew than other Hymenoptera, with rearrangements of the tRNA and protein-coding genes. The A+T content in the measured region of the Shandong clade was 84.4%, the AT skew was 0.1291, and the GC skew was -0.1619.The A+T content in the measured region of Liaoning clade was 84.0%, the AT skew was 0.1305, and the GC skew was -0.1328(Table 3). For comparison, other Hymenoptera have the A+T content range from 76-86% (Wu et al. 2014).

Among the 13 protein-coding genes, the gene with the highest AT content was the *atp8* gene, in both clades, with an AT content of 91.5% for the Shandong clade and 90.9% for the Liaoning clade. Also in both clades, the protein-coding gene with the lowest AT content was the cox1 gene, being 77.4% for the Shandong clade and 77.1%

for the Liaoning clade (Table 4).

There were two ribosomal RNA genes, *rrnL* and *rrnS*. The lengths of the *rrnL* gene were 1359bp (in the Shandong clade) versus 1360bp (Liaoning clade), and for these ribosomal genes, the AT content was 88.0% for the Shandong clade and 88.0% for the Liaoning clade. and the *rrnL* gene occurred in an area between the *trnL1* gene and the *trnA* gene). The *rrnS* gene had 769bp (Shandong clade) compared to 771bp for the Liaoning clade, and the AT content of the *rrnS* gene was 88.8% in the Shandong clade and 89.0% in the Liaoning clade, and this gene occurred in an area between the *trnA* gene and the *trnV* gene.

The length of the tRNA genes of the two clades ranged from 59bp to 75bp in the mitochondrial genome. Three of the 16 tRNA genes, *trnS2*, *trnR* and *trnV*, could not form the normal clover-shaped secondary structure, unlike the other 13 tRNA genes which could do so in both clades.

Hybridization experiment. In both clades, females of *A.mali* that had not mated could parasitize *E. lanigerum* aphids and all offspring were males, indicating neither clade was parthenogenetic

Crosses between the two clades of *A.mali* led to the production of fertile offspring (F1 generation). There were no significant differences in emergence rates or sex ratio (% F) among progeny between clades (Table 5).The relative compatibility of the crosses was greater than 0.75 in both crossing directions (L♂×S♀: 1.188, S♂×L♀: 0.964).

In backcrosses, there were no significant differences in the emergence rates or sex

ratio (% F) of progeny from back crosses between patental clades and the hybrids from crossings of two clades. The relative compatibility values of the two clades were all greater than 0.75(Table 6).

Discussion

We found differences in some morphological characteristics between the two clades of *A.mali* present in China. However, the mitochondrial genomes general structure of the two clades could no[illegible]termine because there were 6 tRNAs that were not detected in both clades. Also, the secondary structures of the *trnS2,trnR,trnV* genes were incomplete, and there were two tRNA mismatches between clades. Crosses between the two clades produced fertileF1females, showing that there were no reproductive barriers between these two geographical populations, and that they have not evolved into two species.

Within species, the body size, developmental duration, and diapause responses of insects frequently vary in different geographical regions (Amy and Nicholas 1999, Sun et al. 2007, Tu et al. 2011). The body size of animals often changes along gradients of latitude or altitude(Bergmann’s rule and the ConverseBergmann's rule (Fairbairn 1997)). Bergmann’s rule postulates that body size of mammals and insects increases with greater latitude (Tu et al. 2011, Fairbairn 1997, Deng and Xue 1997), perhaps because of influences due to photoperiod (Amy and Nicholas 1999). We speculate that the observed difference in body size between the two *A. mali* clades may be due to their occurrence in locations of different latitude(Liaoning c[illegible]e:

122°31'E, 39.20°N, Shandong clade: 116°41'E, 39°91'N).

Mitochondrial genome sequences are an important marker in molecular systematics that are often used in phylogenetic studies to resolve unclear evolutionary relationships among closely related insects (Castro and Dowton 2005, Cha et al. 2007, Sun et al. 2010).

The AT and GC skew values reflect deviation from a population’s base composition, which has important inference value for studying the mechanism of mitochondrial genome replication and transcription (Wei et al. 2010). Skew (AT: Shandong clade 0.1291, Liaoning clade 0.1305; GC: Shandong clade -0.1619, Liaoning clade -0.1328) of the *A. mali* clades were normal and in the general range for wasps. The A+T content of other Hymenoptera is from about 76% to 86% (Wu et al. 2014), which was not significantly different from that of the two clades of *A. mali* (Shandong clade: 84.44%, Liaoning clade: 83.99%), present technology cannot determine the complete sequence of the mitochondrial genome of some insects, including *A. mali* (Du et al.2020). The genetic measures we obtained suggest that the two clades of *A. mali* belonged to the same species.

Crossing experiments are commonly used to resolve species relationships in the parasitic Hymenoptera because laboratory cultures of these wasps are often maintained during biological control projects (Maxwell et al. 2008). According to Pinto, relative reproductive compatibility index values that are below 0.75 suggest partial reproductive isolation among populations (Amy et al. 1999, Sun et al. 2007), Since the reproductive compatibility index values we obtained for the two *A. mali*

clades (L♂×S♀: 1.188, S♂×L♀: 0.964) were far above this 0.75 value, we conclude that these crosses strongly suggest there is no mating incompatibility between these populations, and that there likely is significant gene exchange betwee[illegible]wo clades (Herrero et al. 2018, Fogliata et al. 2016). Indeed, previous studies have documented such gene flow between these populations, which is what would be expected, given the small geographical distance between the two clades' distributions (Zhou et al. 2014). Generally speaking, the formation of species mainly includes the allopatric speciation and the sympatric speciation. Allopatric speciation is also called geographical speciation, which [illegible]sidered by modern evolutionists to be the main way of population speciation into reproductive isolation species (Ayala 1976, Feder and Filchak 1999, Morgan et al. 2000). The process is that before the beginning of speciation, the environment is relatively uniform, the populations exist with a single or a series of similar form. Then, there are environmental differences and subgroups that adapt to each community. However, there was no reproductive isolation.

In this paper, we examined two populations of *A. mali*, the Qingdao population of the Shandong clade and the Qinhuangdao population of the Liaoning clade. We found no isolation between these two populations. However, populations from more widely separated western or southern locations (e.g., in Xinjiang or Yunnan Provinces) of these clades may be more reproductively isolated and this possibility needs further clarification.

Acknowledgements

The authors are highly thankful to Roy Van Driesche for English and Scientific editing as well for suggestions and advice on this research. This work was supported by the National Natural Science Foundation（31772232) and the Taishan Mountain Scholar Constructive Engineering Foundation of Shandong, China.

References

An[illegible], E.A. , and J. G. Nicholas. 1999. Bergmann's rule in the ant lion *Myrmeleon immaculatus*DeGeer (Neuroptera: Myrmeleontidae): geographic variation in body size and heterozygosity. *J. Biogeography* 26: 275-283.

Ayala, F. J. 1976. Genetic differentiation during the speciation process. *Evolutionary Biology* 8:1–75.

Castro, L.R., and M. Dowton. 2005. The position of the Hymenoptera with in the Holometabola as inferred from the mitochondrial genome of *Perga condei* (Hymenoptera: Symphyta: Pergidae). *Mol. Phyl. Evol.* 34(3):469-479.

Cha, S.Y., H. J. Yoon, E.M. Lee, M.H. Yoon, J. S. Hwang, B.R. Jin, Y.S. Han, and I. Kim. 2007. The complete nucleotide sequence and gene organization of the mitochondrial genome of the bumblebee, *Bombus ignitus*(Hymenoptera: Apidae). *Gene* 392(1 -2):206-220.

Chen, W.M., J.N. Yu, Y. Xu, X.L. Liu, and K.Y. Ru. 2006. The first occurrence of woolly apple aphid in Xinjiang. *Xinjiang. Agric. Sci.* 4:309.

Deng, T., and X.X. Xue. 1997.An existing countermeasure of mammals in glacial periods – a new explanation to the Bergmann's Law. *Acta TheriologicaSinica*

17(4):259-265.

Du, L.J., S.J. Wei, X.M. Tan, X.X. Chu, Q.M. Yang, J.Q. Wang, F. H. Wan, and H.X. Zhou. 2020. Mitochondrial genome of *Aphelinus mali* (Haldeman) (Hymenoptera: Aphelinidae) with a higher AT percent and the highest AT-skew in Hymenoptera insects. *Entomol. News* 129(3): 267-285.

Fairbairn, D.J. 1997. Allometry for sexual size dimorphism: pattern and process in the coevolution of body size in males and females. *Annu. Rev. Ecol. Syst.* 28: 659-687.

Feder, J.L. and K.E. Filchak. 1999. It's about time: the evidence for host plant-mediated selection in the apple maggot fly, *Rhagoletis pomonella*, and its implications for fitness trade-offs in phytophagous insects. *Entomol. Exp. Appl.*91(1): 211–225.

Fogliata, S.V., A.Vera, G. Gastaminza, M.I.Cuenya, M.I. Zucchi, E. Willink, A.P. Castagnaro, and M.G. Murúa. 2016. Reproductive isolation between two populations of *Diatraea saccharalis* (F.) (Lepidoptera: Crambidae) from different host plant species and regions in Argentina. *Bull. Entomol. Res.* 106(5): 591-7 (2016).

Herrero, M.I., S.V. Fogliata, A. Vera, A. Casmuz, G.D. Sosa, A.P. Castagnaro, G. Gastaminza, and M.G. Murúa. 2018. Biological characterization and mating compatibility of *Helicoverpa gelotopoeon* (D.) (Lepidoptera: Noctuidae) populations from different regions in Argentina. *Bull. Entomol. Res.* 108(1):108-115.

Jaume, L., A. Simó, G. Ferran, S. José, and A. Georgina. 2015. Woolly apple aphid *Eriosoma lanigerum* Hausmann ecology and its relationship with climatic variables and natural enemies in Mediterranean areas. *Bull. Entomol. Res.* 105(1):60–69.

Kuang, R.P., F. Shan, Y.Z. Tang, and N. Zhong.1985. Evaluation of natural enemies of woolly apple aphid, *Aphelinus mali*(Hald.). *Natural Enemies of Insects*.11(2): 51-56.

Long, C.D., Y.P. Wang, P.Z. Tang.1960. Studies on the biological characteristics and utilization of *Aphelinus mali* Haldeman. *Acta Entomol. Sinica* 10(1): 1-39.

Ma, M., J.Y. Guo, X.M. Tan, L.N. Sun, H.X. Zhou, and F.H. Wan. 2008. *Aphelinus mali* – parasitic wasps with potential to control *Eriosoma lanigerum*. *Entomol. J. East. China*.17(1): 72-73.

Maxwell, K.B., W.K. Susan, A.W. Robert, A.O. William, D.W. David, and A.C. Millicent.2008. Cross mating studies among five fruit fly parasitoid populations: potential biological control implications for tephritid pests. *BioControl* 53:709–724.

Morgan, D.J.W., S.R. Reitz, P.W. Atkinson, and J.T. Trumble.2000. The resolution of Californian population of *Liriomyza huidobrensis* and *Liromyza trifolii*(Diptera: Agromyzidae) using PCR. *Heredity* 85(1),53–61.

Pinto, J.D., R. Stouthamer, G.R. Planter, and E.R. Oatman.1991. Variation in reproductive compatibility in *Trichogramma* and its taxonomic significance (Hymenoptera: Trichgrammatidae). *Ann. Entomol. Soc. Am.* 84(1): 37-46.

Su, M., X.M. Zheng, X.M. Tan, D. Chu, H.X. Zhou, and F.H. Wan.2016. Antennae characteristics of *Aphelinus mali*(Haldeman) male and female adults. *Shandong Agri. Sci.* 48(7): 106-109.

H.X[illegible]hou, and F.H. Wan.2017. Relative efficacy of two clades of *Aphelinus mali* (Hymenoptera: Aphelinidae) for control of woolly apple aphid (Hemiptera: Aphididae) in China. *J. Econ. Entomol.* 110: 35–40.

Su, M., X.M. Tan, Q.M. Yang, C.M. Zhao, F.H. Wan, and H.X. Zhou.2018. Laboratory comparison of two *Aphelinus mali* clades for control of woolly apple aphid from Hebei Province, China. *Bull. Entomol. Res.* 108(3): 400-405.

Shi, Z.H., S.S. Liu, and F.B. Gebremeskel.2002. Comparative study on reproductive compatibility of two geographic populations of *Diadromus collaris*(Gravenhors) (Hym.:Ichneumonidae),a pupal parasitoid of *Plutella xylostella*(L.) (Lep.:Plutellidae). *J. Zhejiang. Univ.* 28(2): 136-140.

Sun, L., H.M. He, and F.S. Xue. 2007. Geographic variation of diapause in insects. *Acta Agriculturae Universitatis Jiangxiensis* 29(6): 921-927.

Sun, Z., J. Zhang, R. Wang, Y.J. Xu, and D.Q. Zhang. 2010. Progress of insect mitochondrial genome. *J. Inspection and Quarantine.* 20(03): 69-73.

Tu, X.Y., Q.W. Xia, C. Chen, Y.S. Chen, X.J. Kuang, and F.S. Xue. 2011. Geographic variation in body weight and size of the Asian corn borer, *Ostrinia furnacalis* Guenée (Lepidoptera: Pyralidae). *Acta. Entomol.Sinica.* 54(2): 143-148.

Wei, S.J., M. Shi, X.X. Chen, M.J. Sharkey, C.V. Achterberg, C.Y. Ye, and J.H. He. 2010. New views on strand asymmetry in insect mitochondrial genomes. *PloS ONE*.5:1767-1780.

Wu, Q.L., Q. Li, Y. Gu, and B.C. Shi. 2014. The complete mitochondrial genome of *Taeniogonalos taihorina* (Bischoff) (Hymenoptera:Trigonalidae) reveals a novel gene rearrangement pattern in the Hymenoptera. *Gene*.543: 76–84.

Yang, Q.R., Z.M. Gao, Y. Zheng, and T. Wang. 2005. Investigation on the natural enemies of woolly apple aphid. *Plant Quarantine*.19(1): 52-52.

Zhang, Q., and W.C. Luo. 2002.Occurrence characteristics of *Eriosoma lanigerum* and its control methods. *Entomol. Knowledge*. 39(5): 340–342.

Zhang, R.M., H.X. Zhou, D. Guo, Y.L. Tao, and F.H. Wan. 2014. Two putative bridgehead populations of *Aphelinus mali* (Hymenoptera: Aphelinidae) introduced in China. *FloridaEntomol*. 97(2): 401-405.

Zhou, H.X., Y. Yu, X.M. Tan, A.D. Chen, and J.G. Feng. 2014. Biological control of insect pests in apple orchards in China. *Biol. Contr*.68:47–56.

Zhou, H.X., R.M. Zhang, D. Guo, Y.L. Tao, F.H. Wan, Q. Wu, and D. Chu. 2014. Analysis of genetic diversity and structure of two clades of *Aphelinus mali*(Hymenoptera: Aphelinidae) in China. *Florida.Entomol*. 97(2): 699–706.

Table1Morphological measurements of two clades of *Aphelinus mali* in China. Data are mean ± SD(μm), and different letters in the same row indicate significant differences.

Character length		Females (Mean ± SD)		Males (Mean ± SD)	
		Shandong	Liaoning	Shandong	Liaoning
Whole body		810.93 ± 16.03b	870.29 ± 12.18a	679.34 ± 12.34d	758.38 ± 15.95c
Abdomen		384.12 ± 9.75b	424.43 ± 7.87a	292.96 ± 7.53d	339.94 ± 8.04c
Fore wing		682.40 ± 11.09c	770.86 ± 9.39a	609.84 ± 8.42d	735.96 ± 8.35b
Hind wing		303.46 ± 4.99c	336.84 ± 4.14a	277.70 ± 4.02d	321.52 ± 3.51b
Anterior leg	Femur	176.70 ± 3.37c	195.25 ± 2.52a	167.46 ± 2.14d	188.30 ± 2.24b
	Tibia	147.32 ± 2.67b	163.43 ± 2.29a	138.67 ± 2.20c	159.98 ± 2.36a
	Tarsus	158.73 ± 2.87b	168.58 ± 2.87a	150.56 ± 2.18c	166.04 ± 2.48a
	Pre-tarsus	19.36 ± 0.41b	24.14 ± 1.36a	19.54 ± 0.46b	23.31 ± 0.38a
	Spur	26.23 ± 0.96bc	27.56 ± 0.56b	25.72 ± 0.71c	29.17 ± 0.76a
	Total leg	528.32 ± 8.42b	551.39 ± 6.96a	476.22 ± 6.33c	543.33 ± 9.01a
Middle leg	Femora	214.75 ± 3.14c	238.31 ± 3.11a	200.52 ± 3.22d	228.08 ± 2.85b
	Tibia	249.91 ± 4.53c	276.08 ± 3.51a	229.60 ± 3.50d	265.51 ± 3.05b
	Tarsus	202.98 ± 3.74b	215.30 ± 3.24a	191.74 ± 2.87c	210.43 ± 3.03a
	Pre-tarsus	19.36 ± 0.41b	23.26 ± 0.29a	19.87 ± 0.42b	22.82 ± 0.29a
	Spur	66.91 ± 1.67b	74.05 ± 1.15a	66.27 ± 1.40b	72.19 ± 2.87a
	Total leg	753.51 ±12.23a	752.95 ± 9.44a	641.73 ± 9.09c	728.55 ± 10.62b
Hind leg	Femora	253.72 ± 4.56a	256.15 ± 3.75a	223.63 ± 3.44c	239.85 ± 2.87b
	Tibia	259.67 ± 4.06c	288.79 ± 4.02a	231.29 ± 3.68d	274.67 ± 3.75b
	Tarsus	226.86 ± 4.23b	242.70 ± 3.72a	211.99 ± 3.41c	233.09 ± 3.30b
	Pretarsus	19.79 ± 0.42b	23.30 ± 0.34a	19.64 ± 0.44b	23.24 ± 0.32a
	Spur	32.20 ± 1.16c	36.54 ± 0.73a	28.60 ± 0.92d	34.16 ± 0.68b
	Total leg	792.33 ± 12.32a	810.94 ± 11.57a	686.55 ± 9.61c	768.52 ± 12.10b
Antenna	Funicle1	16.52 ± 0.44b	19.03 ± 0.31a	16.27 ± 0.43b	18.96 ± 0.32a
	Funicle2	18.04 ± 0.30b	19.33 ± 0.30a	16.44 ± 0.29c	18.94 ± 0.30a
	Funicle3	33.12 ± 0.44c	36.57 ± 0.54b	46.83 ± 0.76a	45.18 ± 1.52a
	Club	91.13 ± 1.37d	106.76 ± 1.03b	100.10 ± 2.19c	113.90 ± 1.72a
	Total antenna	158.81 ± 2.18c	181.68 ± 1.68b	179.62 ± 2.92b	212.50 ± 2.55a

Table 2 Comparisons of the mitochondrial genome between two clades of *Aphelinus mali* in China

Gene	Clade	Strand	Length(bp）	Anti/start codon	Stop codon	Intergenic nucleotides
cox3	Shandong	-	732	ATG	TAA	39
	Liaoning	-	732	ATG	TAA	2
atp6	Shandong	-	651	ATT	TAA	3
	Liaoning	-	651	ATT	TAG	-10
atp8	Shandong	-	156	ATT	TAA	-10
	Liaoning	-	162	ATT	TAA	24
trnD	Shandong	-	68	GTC		24
	Liaoning	-	71	GTC		10
trnK	Shandong	+	70	TTT		9
	Liaoning	+	74	TTT		2
cox2	Shandong	-	660	ATT	TAA	2
	Liaoning	-	660	ATT	TAA	0
trnL2	Shandong	-	68	TAA		0
	Liaoning	-	67	TAA		14
cox1	Shandong	-	1512	ATT	TAA	-18
	Liaoning	-	1509	ATT	TAA	-18
trnE	Shandong	+	67	TTC		4
	Liaoning	+	67	TTC		-1
trnF	Shandong	-	70	GAA		-1
	Liaoning	-	67	GAA		0
nad5	Shandong	-	1479	ATT	TAA	11
	Liaoning	-	1563	ATT	T	12
trnH	Shandong	-	68	GTG		0
	Liaoning	-	68	GTG		0
nad4	Shandong	-	1335	ATG	T	-7
	Liaoning	-	1308	ATG	T	-7
nad4L	Shandong	-	273	ATA	TAA	-7
	Liaoning	-	267	ATA	TAA	-7
trnT	Shandong	+	66	TGT		0
	Liaoning	+	66	TGT		
trnP	Shandong	-	65	TGG		2
	Liaoning	-	64	TGG		
nad6	Shandong	+	519	ATA	T	15
	Liaoning	+	513	ATA	T	1

Gene	Clade	Strand	Length(bp）	Anti/start codon	Stop codon	Intergenic nucleotides
cob	Shandong	+	1083	ATA	TAA	1
	Liaoning	+	1083	ATA	TAA	27
trnS2	Shandong	+	65	TGA		27
	Liaoning	+	65	TGA		73
trnS2	Shandong	+	65	TGA		77
	Liaoning	+	70	TGA		77
trnS2	Shandong	+	65	GGA		78
	Liaoning	+	65	GGA		77
trnS2	Shandong	+	65	TGA		149
	Liaoning	+	65	TGA		149
nad1	Shandong	-	891	ATA	TAA	0
	Liaoning	-	891	ATA	TAA	0
trnL1	Shandong	-	66	TAG		0
	Liaoning	-	66	TAG		0
rrnL	Shandong	-	1359			0
	Liaoning	-	1360			0
trnA	Shandong	-	70	TGC		0
	Liaoning	-	67	TGC		0
rrnS	Shandong	-	769			0
	Liaoning	-	771			0
trnV	Shandong	-	56	TAC		323
	Liaoning	-	56	TAC		20
nad2(p)	Shandong	-	240	ATT	ATT	40
	Liaoning	-	213	ATT	TGA	
trnN	Shandong	+	66	GTT		0
	Liaoning	+	66	GTT		
trnC	Shandong	-	64	GCA		
	Liaoning	-	64	GCA		44
trnR	Shandong	-	59	TCG		
	Liaoning	-	59	TCG		2
nad3	Shandong	-	312	ATA	TAA	
	Liaoning	-	312	ATA	TAA	-3
trnG	Shandong	-	65	TCC		
	Liaoning	-	65	TCC		41
//	Shandong		998			
	Liaoning		972			

Table 3 The base content, AT skewness and GC skewness of the two *Aphelinus mali* clades compared to other Hymenoptera

Species	T%	C%	A%	G%	A+T%	ATskew	GCskew
Aphelinus mali SC	36.69	9.40	47.30	6.61	84.44	0.1291	-0.1619
Aphelinus mali LC	36.69	9.40	47.30	6.61	83.99	0.1305	-0.1328
Taeniogonalos taihorina	43.51	6.77	41.09	8.63	84.60	-0.0286	0.1208
Spathius agrili	45.12	6.55	38.88	9.45	84.00	-0.0743	0.1813
Cotesia vestalis	47.46	5.78	39.70	7.06	87.16	-0.0890	0.0997
Enicospilus sp.	43.44	8.73	41.45	6.08	85.19	-0.0198	-0.1789
Abispa ephippium	39.50	14.60	39.10	6.70	78.60	-0.0051	-0.3709
Bombus hypocrita	42.56	9.68	42.77	4.98	85.33	0.0025	-0.0275
Pergacondei	33.80	14.60	42.80	8.80	76.60	0.1175	-0.2479

Table 4 The base composition of mitochondrial genomic coding gene and rRNA gene of two clades of *Aphelinus mali* in China

Gene	Clades	T%	C%	A%	G%	A+T%	AT skew	GC skew
cox3	Shandong	30.13	12.05	49.23	8.59	79.36	0.2454	-0.1676
	Liaoning	29.87	12.44	49.10	8.59	78.97	0.2435	-0.1831
atp6	Shandong	30.83	8.85	50.88	9.44	81.71	0.2454	-0.0323
	Liaoning	31.56	9.29	50.15	9.00	81.71	0.2282	-0.0159
atp8	Shandong	42.42	4.85	49.09	3.64	91.52	0.0729	-0.1427
	Liaoning	41.82	4.85	49.09	4.24	90.91	0.0800	-0.0671
cox2	Shandong	34.51	11.01	46.26	8.22	80.76	0.1455	-0.1450
	Liaoning	34.80	11.01	45.81	8.37	80.62	0.1366	-0.1362
cox1	Shandong	31.66	12.81	45.75	9.78	77.41	0.1820	-0.1341
	Liaoning	30.82	13.32	46.27	9.59	77.09	0.2004	-0.1628
nad5	Shandong	33.21	.72	52.21	5.85	85.42	0.2224	-0.1968
	Liaoning	33.13	8.49	52.63	5.74	85.77	0.2274	-0.1933
nad4	Shandong	34.28	8.79	50.67	6.26	84.95	0.1929	-0.1681
	Liaoning	32.11	11.60	47.01	9.28	79.12	0.1883	-0.1111
nad4L	Shandong	34.01	8.42	54.55	3.03	88.55	0.2320	-0.4707
	Liaoning	35.35	7.74	54.88	2.02	90.23	0.2164	-0.5860
nad6	Shandong	45.82	6.00	44.91	3.27	90.73	-0.0100	-0.2945
	Liaoning	44.91	6.79	45.06	3.24	89.97	0.0017	-0.3539
cob	Shandong	43.73	10.93	35.29	10.04	79.02	-0.1068	-0.0424
	Liaoning	42.81	11.58	35.53	10.09	78.33	-0.0929	-0.0688
nad1	Shandong	33.76	11.75	47.44	7.05	81.20	0.1685	-0.2500
	Liaoning	33.44	12.39	47.22	6.94	80.66	0.1708	-0.2818
nad3	Shandong	33.33	9.32	52.26	5.08	85.59	0.2212	-0.2942
	Liaoning	33.33	9.60	51.41	5.65	84.75	0.1661	-0.2590
rrnl	Shandong	41.20	7.46	46.76	4.57	87.97	0.0632	-0.2352
	Liaoning	41.08	7.55	46.95	4.42	88.03	0.0667	-0.2615
rrns	Shandong	42.39	7.67	46.42	3.51	88.82	0.0454	-0.3721
	Liaoning	42.15	7.65	46.82	3.37	88.98	0.0525	-0.3884
Nad2	Shandong	35.00	5.00	55.00	5.00	90.00	0.2222	0
	Liaoning	35.92	4.92	54.93	4.23	90.85	0.2092	-0.0754

Table 5 Results of crosses between the two *Aphelinus mali* clades, in terms of the number of pupae, progeny produced, emergence rate, female ratio and relative compatibility of hybridization. Data in the same row are means ± SD, different letters indicate significant differences (*P*<0.05). L: Liaoning clade. S: Shandong clade

	# pupae	# adult progeny	% emergence	Sex ratio (%F)	relative compatibility
L♂×S♀（n=18）	5.28 ± 2.93a	4.67 ± 3.03ab	85.09± 16.75a	0.57 ± 0.28a	1.188
S♂×L♀（n=15）	4.86 ± 4.28a	3.78 ± 3.62a	77.94 ± 28.87a	0.53 ± 0.32a	0.964
S♂×♀（n=15）	7.00 ± 4.95a	6.37 ± 1.69ab	91.00 ± 13.98a	0.48 ± 0.33a	——
L♂×♀（n=18）	8.67 ± 2.89a	7.29 ± 2.39b	84.08± 30.29a	0.55 ± 0.34a	——

Table 6 The number of pupae, progeny produced, emergence rate, sex ratio (as %F)and relative compatibility of backcross. Data i[illegible]e same row are mean ± SD, different letters indicate significant differences (*P*<0.05). L: Liaoning clade; S: Shandong clade.

	[illegible] pupae	# progeny	% emergence	Sexratio (% F)	relative compatibility
（S♀×L♂）♂backcross S♀ （n=5）	3.80 ± 2.95a	3.40 ± 3.88b	77.50 ± 43.66a	0.47 ± 0.35a	0.980
（S♀×L♂）♀backcross L♂ （n=3）	4.33 ± 1.53a	4.00 ± 1.00ab	94.40 ± 9.62a	0.59 ± 0.08a	1.230
（S♂×L♀）♂backcross L♀ （n=3）	3.33 ± 1.53a	3.00 ± 2.00b	83.30 ± 28.87a	0.76 ± 0.21a	1.382
（S♂×L♀）♀backcross S♂ （n=3）	6.67 ± 2.08a	6.33 ± 2.52ab	93.30 ± 11.55a	0.61 ± 0.10a	1.110
S♂×S♀ （n=15）	7.00 ± 4.95a	6.37 ± 1.69ab	91.00 ± 13.98a	0.48 ± 0.33a	——
L♂×L♀ （n=18）	8.67 ± 2.89a	7.29 ± 2.39a	84.08± 30.29a	0.55 ± 0.34a	——

Figure Click here to access/download;Figure;figure.docx

Fig. 1. The linear arrangement of mitochondrial genomes in the sequences of two clades

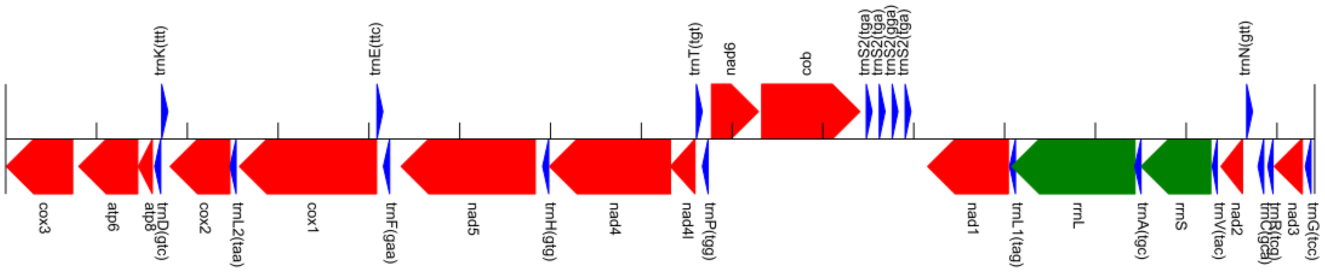

Shandong clade

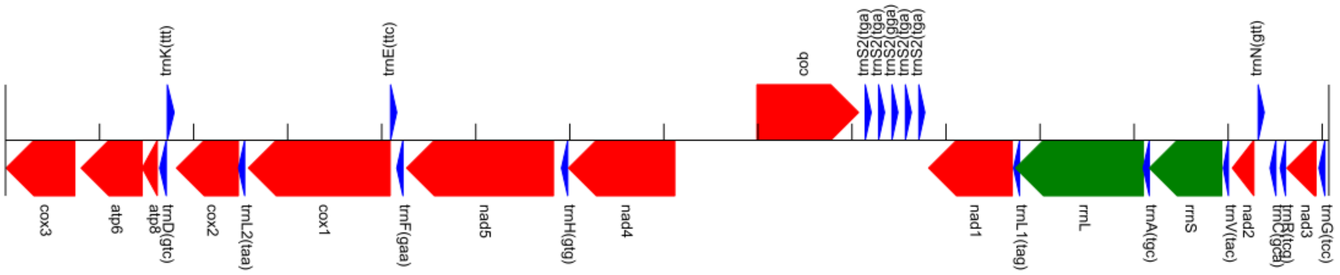

Liaoning clade
