# Supplementary material for: Differences in morphology, mitochondrial genomes, and reproductive compatibility between two clades of parasitic wasps Aphelinus mali (Hymenoptera: Aphelindae) in China
Source: PLoS One. 2023 Feb 2;18(2):e0279663. doi: 10.1371/journal.pone.0279663 (PMC9894431; doi:10.1371/journal.pone.0279663)
Supplement: Attachment — Submitted filename: PONE-D-21-21080_R1_reviewer comments.pdf [file pone.0279663.s009.pdf]

PLOS ONE

Differences in morphology, mitochondrial genomes, and reproductive compatibility between two clades of parasitic wasps Aphelinus mali (Hymenoptera: Aphelindae) in China

--Manuscript Draft--

**Manuscript Number:**	PONE-D-21-21080R1
**Article Type:**	Research Article
**Full Title:**	Differences in morphology, mitochondrial genomes, and reproductive compatibility between two clades of parasitic wasps Aphelinus mali (Hymenoptera: Aphelindae) in China
**Short Title:**	Differences between two clades of Aphelinus mali (Hemiptera: Aphididae) in China
**Corresponding Author:**	Hongxu Zhou<br>Qingdao Agricultural University<br>Qingdao, Shandong CHINA
**Keywords:**	Aphelinus mali, hybridization, morphological characteristics, mitochondrial genome
**Abstract:**	Aphelinus mali (Haldeman) (Hymenoptera: Aphelinidae) in China is comprised of two clades (termed, the Shandong and Liaoning clades). In order to clarify the genetic relationship, between these two clades, we compared and analyzed the morphological characteristics and the mitochondrial genome of each, and performed a hybridization experiment. Morphological results showed that both males and females of the Liaoning clade were larger than in the Shandong clade, in terms of whole body, abdominal, wing and antennal lengths, but there were no significant differences between clades for total length of the middle or hind leg of females. The length of the mitochondrial genome of the Shandong clade was14415 bp and, for the Liaoning clade, it was 14804 bp. Each contained 31 genes, including 13 protein-encoded genes, 16 tRNA genes, and 2 rRNA genes. The highest AT level among the 13 protein-coding genes for the two clades were the same gene (ATP8) (Shandong clade, 91.52%; Liaoning clade, 90.91%). By hybridization and backcrossing, we found there was no cross incompatibility between these two clades of A. mali . Our results indicate that the historic geographical isolation between these clades has not yet caused reproductive isolation of these populations, and they belong to the same species.
**Order of Authors:**	Min Su
	Lingjun Du
	Yasir Ali Muhammad
	Jianing Yu
	Mengyu Chi
	Ziwen Teng
	Yinjun Fan
	Xiumei Tan
	Hongxu Zhou
**Response to Reviewers:**	The comments that reviewers and editor have been revised, and we submit as an attachment.
**Additional Information:**	
**Question**	**Response**
**Financial Disclosure**<br><br>Enter a financial disclosure statement that describes the sources of funding for the	National Natural Science Foundation (31772232) .<br>Taishan Mountain Scholar Constructive Engineering Foundation of Shandong, China<br><br>The funder "National Natural Science Foundation (31772232)" play a role in study

work included in this submission. Review the submission guidelines for detailed requirements. View published research articles from *PLOS ONE* for specific examples.<br><br>This statement is required for submission and **will appear in the published article** if the submission is accepted. Please make sure it is accurate.<br><br>**Unfunded studies**<br>Enter: *The author(s) received no specific funding for this work.*<br><br>**Funded studies**<br>Enter a statement with the following details:<br>• Initials of the authors who received each award<br>• Grant numbers awarded to each author<br>• The full name of each funder<br>• URL of each funder website<br>• Did the sponsors or funders play any role in the study design, data collection and analysis, decision to publish, or preparation of the manuscript?<br>• **NO** - Include this sentence at the end of your statement: *The funders had no role in study design, data collection and analysis, decision to publish, or preparation of the manuscript.*<br>• **YES** - Specify the role(s) played.<br><br>* typeset	design, data collection and preparation of the manuscript, the funder " Taishan Mountain Scholar Constructive Engineering Foundation of Shandong, China " play a role in data collection.
**Competing Interests**<br><br>Use the instructions below to enter a competing interest statement for this submission. On behalf of all authors, disclose any competing interests that could be perceived to bias this work—acknowledging all financial support and any other relevant financial or non-financial competing interests.<br><br>This statement is required for submission and **will appear in the published article** if the submission is accepted. Please make sure it is accurate and that any funding sources listed in your Funding Information	The authors have declared that no competing interests exist.

later in the submission form are also declared in your Financial Disclosure statement.<br><br>View published research articles from *PLOS ONE* for specific examples.<br><br>**NO authors have competing interests**<br><br>Enter: *The authors have declared that no competing interests exist.*<br><br>**Authors with competing interests**<br><br>Enter competing interest details beginning with this statement:<br><br>*I have read the journal's policy and the authors of this manuscript have the following competing interests: [insert competing interests here]*<br><br>* typeset	
**Ethics Statement**<br><br>Enter an ethics statement for this submission. This statement is required if the study involved:<br><br>• Human participants<br>• Human specimens or tissue<br>• Vertebrate animals or cephalopods<br>• Vertebrate embryos or tissues<br>• Field research<br><br>Write "N/A" if the submission does not require an ethics statement.<br><br>General guidance is provided below. Consult the submission guidelines for detailed instructions. **Make sure that all information entered here is included in the Methods section of the manuscript.**	N/A

Powered by Editorial Manager® and ProduXion Manager® from Aries Systems Corporation

**Format for specific study types**<br><br>**Human Subject Research (involving human participants and/or tissue)**<br>• Give the name of the institutional review board or ethics committee that approved the study<br>• Include the approval number and/or a statement indicating approval of this research<br>• Indicate the form of consent obtained (written/oral) or the reason that consent was not obtained (e.g. the data were analyzed anonymously)<br><br>**Animal Research (involving vertebrate animals, embryos or tissues)**<br>• Provide the name of the Institutional Animal Care and Use Committee (IACUC) or other relevant ethics board that reviewed the study protocol, and indicate whether they approved this research or granted a formal waiver of ethical approval<br>• Include an approval number if one was obtained<br>• If the study involved *non-human primates*, add *additional details* about animal welfare and steps taken to ameliorate suffering<br>• If anesthesia, euthanasia, or any kind of animal sacrifice is part of the study, include briefly which substances and/or methods were applied<br><br>**Field Research**<br><br>Include the following details if this study involves the collection of plant, animal, or other materials from a natural setting:<br>• Field permit number<br>• Name of the institution or relevant body that granted permission	
**Data Availability**<br><br>Authors are required to make all data underlying the findings described fully available, without restriction, and from the time of publication. PLOS allows rare exceptions to address legal and ethical concerns. See the PLOS Data Policy and FAQ for detailed information.	Yes - all data are fully available without restriction

A Data Availability Statement describing where the data can be found is required at submission. Your answers to this question constitute the Data Availability Statement and **will be published in the article**, if accepted.<br><br>**Important:** Stating 'data available on request from the author' is not sufficient. If your data are only available upon request, select 'No' for the first question and explain your exceptional situation in the text box.<br><br>Do the authors confirm that all data underlying the findings described in their manuscript are fully available without restriction?	
**Describe where the data may be found in full sentences. If you are copying our sample text, replace any instances of XXX with the appropriate details.**<br><br>• If the data are **held or will be held in a public repository**, include URLs, accession numbers or DOIs. If this information will only be available after acceptance, indicate this by ticking the box below. For example: *All XXX files are available from the XXX database (accession number(s) XXX, XXX.*).<br>• If the data are all contained **within the manuscript and/or Supporting Information files**, enter the following: *All relevant data are within the manuscript and its Supporting Information files.*<br>• If neither of these applies but you are able to provide **details of access elsewhere**, with or without limitations, please do so. For example:<br><br>*Data cannot be shared publicly because of [XXX]. Data are available from the XXX Institutional Data Access / Ethics Committee (contact via XXX) for researchers who meet the criteria for access to confidential data.*<br><br>*The data underlying the results presented in the study are available from (include the name of the third party*	All relevant data are within the manuscript and its Supporting Information files.

and contact information or URL).<br>• This text is appropriate if the data are owned by a third party and authors do not have permission to share the data.<br><br>* typeset	
Additional data availability information:	

Manuscript
Click here to access/download;Manuscript;11-14 unmarked version.docx

Differences in morphology, mitochondrial genomes, and reproductive compatibility between two clades of parasitic wasps *Aphelinus mali* (Hymenoptera: Aphelindae) in China

Min Su[a,b], Lingjun Du[a], Yasir Ali Muhammad[a], Jianing Yu[a], Mengyu Chi[a], Ziwen Teng[a], Yinjun Fan[a], Xiumei Tan[a], Hongxu Zhou[a,*]

[a]College of Botanical Medicine, Qingdao Agricultural University, Key Laboratory of Integrated Plant Disease and Pest Control, China-Australia Joint Institute of Agricultural and Environmental Health, Qingdao 266109, China (18765903010@163.com; 1163355473@qq.com; m.yasirali4663@gmail.com; 2354846762@qq.com; 1538848218@qq.com; tzwbat@126.com; fanyinjun89@126.com; xmtanly@163.com; hxzhouqd@sina.cn)

[b]Agricultural and rural Bureau of Zhangqiu District, Jinan 250200, China (18765903010@163.com)

* Corresponding author(hxzhouqd@sina.cn)

Min Su helped write the article; Lingjun Du contributed the work on the mitochondrial genomes; Jianing Yu contributed to the part on reproductive compatibility; Mengyu Chi contributed to the part on morphological characteristics; Yasir Ali Muhammad, Ziwen Teng, Yinjun Fan, Xiumei Tan and Hongxu Zhou checked the manuscript.

Abstract

Aphelinus mali (Haldeman) (Hymenoptera: Aphelinidae) in China is comprised of two clades (termed, the Shandong and Liaoning clades). In order to clarify the genetic relationship, between these two clades, we compared and analyzed the morphological characteristics and the mitochondrial genome of each, and performed a hybridization experiment. Morphological results showed that both males and females of the Liaoning clade were larger than in the Shandong clade, in terms of whole body, abdominal, wing and antennal lengths, but there were no significant differences between clades for total length of the middle or hind leg of females. The length of the mitochondrial genome of the Shandong clade was14415 bp and, for the Liaoning clade, it was 14804 bp. Each contained 31 genes, including 13 protein-encoded genes, 16 tRNA genes, and 2 rRNA genes. The highest AT level among the 13 protein-coding genes for the two clades were the same gene (*ATP8*) (Shandong clade, 91.52%; Liaoning clade, 90.91%). By hybridization and backcrossing, we found there was no cross incompatibility between these two clades of *A. mali*. Our results indicate that the historic geographical isolation between these clades has not yet caused reproductive isolation of these populations, and they belong to the same species.

Key words *Aphelinus mali*, hybridization, morphological characteristics, mitochondrial genome

Introduction

Woolly apple aphid, *Eriosoma lanigerum* (Hausmann) (Hymenoptera: Aphididae), is an important quarantine pest of apples in many parts of the world[1]. In recent years, the damage caused by this aphid has become a serious problem in China, surveys in Rizhao, Shandong Province from 2000 to 2002, found WAA in about 8000 hectares of orchard, with 10–20% of the trees being infested, causing an annual loss of 5 × 106 kg of apples (Wang et al., 2011), and the pest range has continued to increase[2-4]. The endoparasitoid *Aphelinus mali* (Haldeman) (Hymenoptera: Aphelinidae) is the dominant natural enemy of *E. lanigerum* in China[5]and is considered the most effective of the aphid's biocontrol agents[6,7]. *Aphelinus mali* has been introduced into 51 countries and has established in 42 of them[8].

Aphelinus mali was introduced into China twice: the first introduction was from Japan into Dalian and Lvshun, Liaoning Province (122°31'E, 39.20°N) in 1942, while the second was from the former Soviet Union in 1950 into Qingdao, Shandong Province (116°41'E, 39°91'N) [9]. High intra-specific variability is expected when a species has a large geographic distribution, as does *A. mali*[10]. Based on the study of the mitochondrial COI gene of this parasitoid, *A. mali* in China is comprised of two regional clades (named the Shandong and Liaoning clades) [11,12](Fig.1). The *A. mali* population in Qingdao and Tai'anin Shandong province and the population in Dalian and Huludao in Liaoning province differ in their biological characteristics and control potential[13,14].

The study of the genetic relationship between these two clades can help to assess

the ecological adaptability of each clade and determine whether they have undergone intra-specific differentiation. Such information can provide new ideas and methods for the introduction, reproduction, and field release of *A.mali* as a biological control agent against *E. lanigerum*. The morphological characteristics and mitochondrial genome were compared to clarify the genetic relationships between two clades of *A.mali*. The experimenters also performed a hybridization experiment to see if any reproduction isolation has developed between these clades.

Materials and Methods

Morphological comparison of clades. To obtain parasitoids for morphological comparisons, *E. lanigerum* aphids were collected from apple orchards in Tai'an (117°13E, 36°19N), Shandong province and Dalian (121°52E, 38°95N), Liaoning province, in mid-August, 2014. Parasitized aphids which black and mummified were noted and placed in Petri dishes and held at 25°C for parasitoid emergence. Adults of *A. mali* were removed every day, placed in 100% ethanol, and conserved at 20°C. Male and female *A. mali* were separated based on the larger body size of females, antennal shape (the third section of the female antenna at the funicular joint is square, while that of the male is rectangular)[15], and the abdominal shape (females have a short, thick abdomen, while the male abdomen is slender)[9].

Images of body regions or parts were taken with the universal video imaging system (LY-WN-HPCCD (10),Chengdu Li Yang Precision Mechanical and Electrical Co., Ltd., China). We measured the length of the body and abdomen, the length and width of front and hind wing, the length of the whole leg, the tibia, tarsae, anterior

tarsal segment, and calcar (spine at the tip of the tibia) of the anterior, middle, and hind legs, the length and width of the rod section of the antennal, and the first, the second, and the third antennal segments. One hundred adults of *A. mali* (50M and 50F) from each sample area were measured.

Mitochondrial genome sequencing. For mitochondrial sequencing, aphids parasitized by *A. mali* were collected from one abandoned apple orchard in Tai'an, Shandong Province and another in Huludao (120°87E, 40°77N), Liaoning province. At each site, the five-point sampling method was used, sampling ten trees at each of the five points. On each of these 50 trees per location, five branches bearing blackened *E. lanigerum* aphids that were parasitized by *A. mali* were collected and put into a 25°C incubator in the laboratory to await *A. mali* emergence. One hundred adults of *A. mali* were obtained from each site and placed individually in 1.5 ml centrifuge tubes containing absolute ethanol and sent to Wuhan Transduction Bio Co., LTD for sequencing.

Primers designed to match generally conserved regions of target mtDNA were used to amplify short fragments from *COX3, ATP6, COX1, NAD5, NAD4, CYTB, NAD1, RRNL, RRNS* (Table 1). Specific primers were designed based on these conserved regions sequences and used to amplify the remained mtDNA sequence in several PCR reactions. Primers were designed to produce amplicons with overlaps of about 100 bp. The PCR reaction was performed using the LA Taq polymerase. The PCR conditions were as follows: initial denaturation 94°C for 2 min, then 35 cycles of denaturation at 94°C for 30s, annealing at 55°C for 30s, and extension at 72°C for 1 min/kb, followed

by the final extension at 72°C for 10 min. The total volume for PCR and LA-PCR was 50 μL, of which 0.5 μL Takara LATaq (5 U/μL), 5 μL10×LATaq Buffer II (Mg^{2+}), 8 μLdNTP Mixture (2.5 mM), 60 ng Template, and the total volume was then made up with distilled water. The final concentration of the forward and reverse primers was 0.2~1.0 μM, and that of $MgCl_2$ was 2.0 mM. The PCR products were sequenced directly, or if needed first cloned into a pMD18-T vector (Takara, JAP) and then sequenced, by the dideoxynucleotide procedure, using an ABI 3730 automatic sequencer (Sanger sequencing) using the same set of primers. All obtained fragments were quality-proofed (electropherogram) and BLASTed to confirm that the amplicon is the actual target sequence. Whenever the quality was sub-optimal, sequencing was repeated. All obtained fragments were BLASTed to confirm that the amplicon is the target sequence. Mitogenome was assembled stepwise with the help of DNAstar v7.1 program, making sure that the overlaps were identical, and that no NUMTS were incorporated into the sequence.

Cross mating experiment. For the cross-mating experiment, *A.mali* as parasitized aphids on apple trees (as described below) were collected from Qingdao (120°39E, 36°31N) to represent the Shandong clade and from Qinhuangdao (119°48E, 39°83N) from the Liaoning clade.

The experimenters choose an apple orchard with less management and protected the apple trees that had colonies of *E. lanigerum* with screen cages (1m×1m×2m). Thereafter, the parasitized black aphids were eliminated daily until it didn't appear, made sure there were no *A. mali* in the *E. lanigerum* colony, and then kept it in its

natural state in the apple orchard, waiting for parasitized by the *A.mali* in our experiment.

Crosses and reciprocal crosses between the two populations (one per clade) were made using within clade crosses in each population as the control group (Liaoning♀×Shandong♂, Liaoning♂×Shandong♀); all crosses were repeated 15-20 times. The *A. mali* were held in the Petri dishes (13.5 cm diameter, had cotton ball with 10% honey water) at 25℃, 70% RH, and a 16:8 h L:D photoperiod. For each cross, we recorded the number of hosts attacked by each mated, crossed female and the number of offspring, and these offspring's sex ratio (as % female).

The F1 generation of each hybrid cross were backcrossed with the parents of each population, conducting a "within hybrid (F1) population" cross as the control group, with 3-5 replicates per type of crossing. We recorded the number of parasitoid offspring for each crossed female, for all crossing combinations, as well as the mean sex ratio (MSR) of the resulting backcrossed progeny (as % female). The relative compatibility of an inter-group crossing (A x B) was expressed as[16,17]:

$$\frac{\mathrm{MSR}(\mathrm{A}♀ \times \mathrm{B}♂)}{\mathrm{MSR}(\mathrm{A}♀ \times \mathrm{A}♂)} \times 100\%$$

Statistical analysis. Morphological measurements as well as the hybridization and backcross rates are presented as means ± standard deviations (SD), calculated using Statistical Product and Service Solutions (SPSS) 19.0. Significant differences were determined using one-way analysis of variance (ANOVA) corrected by SPSS 19.0. The independent samples t-test was used to analyze the data on parthenogenesis between two clades, using SPSS 19.0.

For genomic analysis, we used MITOS Webserver (http:// mitos. bioinf. uni-leipzig. de/index. py) to annotate and analyze the mitochondrial genome sequence and obtain the linear alignment of the mitochondrial genome sequence. The AT-skew and GC-skew were calculated using DNASTAR as follows: AT-skew = (A% -T%)/ (A% +T%) and GC-skew = (G%-C %)/ (G%+C%).

Results

Comparison of morphological characters. Measurements of body dimensions (Table 2) showed that both males and females from the Liaoning clade were bigger than those of the Shandong clade (Fig.2),in terms of the whole body, abdominal, wing, and antennal segment lengths. But the total length of the middle and hind legs showed no significant differences in females between two clades.

Mitochondrial genome sequencing and analysis. The mitochondrial genome had 14415 bp for the Shandong clade and 14804 bp for Liaoning. Both clades contained 31 genes, including 13 protein-encoded genes, 16 tRNA genes, and 2 rRNA genes (Table 3, Fig.3). The unmeasured area was in the A+T-rich region, which usually contains six tRNA genes (*TRNY, TRNM, TRNI, TRNQ, TRNW,* and *TRNS1*) in such genomes, but which were absent in *A. mali*. Genomes of both clades had a higher A+T content and AT skew than other Hymenoptera, with rearrangements of the tRNA and protein-coding genes. The A+T content in the measured region of the Shandong clade was 84.44%, the AT skew was 0.1291, and the GC skew was -0.1619. The A+T content in the measured region of Liaoning clade was 83.99%, the AT skew was 0.1305, and the GC skew was -0.1328 (Table 4).For comparison, other Hymenoptera

have the A+T content range from 76-86%[18].

Among the 13 protein-coding genes, the gene with the highest AT content was the *ATP8* gene, in both clades, with an AT content of 91.52% for the Shandong clade and 90.91% for the Liaoning clade. Also in both clades, the protein-coding gene with the lowest AT content was the cox1 gene, being 77.41% for the Shandong clade and 77.09% for the Liaoning clade (Table 5).

There were two ribosomal RNA genes, *RRNL* and *RRNS*. The lengths of the *RRNL* gene were 1359bp (in the Shandong clade) versus 1360bp (Liaoning clade), and for these ribosomal genes, the AT content was 87.97% for the Shandong clade and 88.03% for the Liaoning clade,and the *RRNL* gene occurred in an area between the *TRNL1* gene and the *TRNA* gene. The *RRNS* gene had 769bp (Shandong clade) compared to 771bp for the Liaoning clade, and the AT content of the *RRNS* gene was 88.82% in the Shandong clade and 88.98% in the Liaoning clade, and this gene occurred in an area between the *TRNA* gene and the *TRNV* gene.

The length of the tRNA genes of the two clades ranged from 59bp to 75bp in the mitochondrial genome. Three of the 16 tRNA genes, *TRNS2*, *TRNR* and *TRNV*, could not form the normal clover-shaped secondary structure, unlike the other 13 tRNA genes which could do so in both clades.

Hybridization experiment. In both clades, unmated *A.mali* females could parasitize *E. lanigerum* aphids and all offsprings were males, indicating neither clade was parthenogenetic.

Crosses between the two clades of *A.mali* led to the production of fertile offsprings

(F1 generation). There were no significant differences in emergence rates or sex ratio (% F) among progeny between clades (Table 6).The relative compatibility of the crosses was greater than 0.75 in both crossing directions (L♂×S♀: 1.188, S♂×L♀: 0.964).

In backcrosses, there were no significant differences in the emergence rates or sex ratio (% F) of progeny from back crosses between patental clades and the hybrids from crossings of two clades. The relative compatibility values of the two clades were all greater than 0.75 (Table 7).

Discussion

We found differences in some morphological characteristics between the two clades of *A.mali* present in China. Because there were 6 tRNAs that were not detected in both clades, the mitochondrial genomes general structure of the two clades could not be determined and compared. Also, the secondary structures of the *TRNS2, TRNR, TRNV* genes were incomplete, and there were two tRNA mismatches between clades. Crosses between the two clades produced fertile F1 females, showing that there were no reproductive barriers between these two geographical populations, and that they have not evolved into two species.

Within species, the body size, developmental duration, and diapause responses of insects frequently vary in different geographical regions[19-21]. The body size of animals often changes along gradients of latitude or altitude (Bergmann’s rule and the ConverseBergmann's rule[22]). Bergmann’s rule postulates that body size of

mammals and insects increases with greater latitude[21-23], perhaps because of photoperiod influences[19]. We speculate that the observed difference in body size between the two *A. mali* clades may be due to their occurrence in locations of different latitude.

Mitochondrial genome sequences are an important marker in molecular systematics that are often used in phylogenetic studies to resolve unclear evolutionary relationships among closely related insects[24-26]. The AT and GC skew values reflect deviation from a population’s base composition, which has important inference value for studying the mechanism of mitochondrial genome replication and transcription[27]. Skew values (AT: Shandong clade 0.1291, Liaoning clade 0.1305; GC: Shandong clade -0.1619, Liaoning clade -0.1328) of the *A.mali* clades were normal and in the general range for wasps[18]. The A+T content of other Hymenoptera is from about 76% to 86%[18], and the values of these two clades of *A.mali* (Shandong clade: 84.44%, Liaoning clade: 83.99%) were within the range. Present technology cannot determine the complete sequence of the mitochondrial genome of some insects, including *A.mali*[28]. The genetic measures we obtained suggest that the two clades of *A. mali* belonged to the same species.

Crossing experiments are commonly used to resolve species relationships in the parasitic Hymenoptera because laboratory cultures of these wasps are often maintained during biological control projects[10]. According to Pint[illegible]6],relative reproductive compatibility index values that are below 0.75 suggest partial reproductive isolation among populations[19,20], since the productive compatibility

index values we obtained for the two *A. mali* clades (L♂×S♀: 1.188, S♂×L♀: 0.964) were far above this 0.75 value, we conclude that these crosses strongly suggest there is no mating incompatibility between these populations, and that there likely is significant gene exchange between the two clades[29,30]. Indeed, previous studies have documented such gene flow between these populations, which is what would be expected, given the small geographical distance between the two clades' distributions[12]. Generally speaking, the formation of species mainly includes the allopatric speciation and the sympatric speciation. Allopatric speciation is also called geographical speciation, which is considered by modern evolutionists to be the main way of population speciation into reproductive isolation species[31-33]. Before speciation, the environment is relatively uniform, the populations exist with a single or a series of similar form. Then, there are environmental differences and subgroups that adapt to each community. However, there was no reproductive isolation.

In this paper, we examined samples from two populations of *A. mali*, the Qingdao population of the Shandong clade and the Qinhuangdao population of the Liaoning clade. We found no reproductive isolation between these two populations. However, populations from more widely separated western or southern locations (e.g., in Xinjiang or Yunnan Provinces) of these clades may be more reproductively isolated and this possibility needs further clarification.

Acknowledgements

The authors are highly thankful to Roy Van Driesche for English and Scientific editing as well for suggestions and advice on this research. This work was supported by the National Natural Science Foundation (31371994) and the Taishan Mountain Scholar Constructive Engineering Foundation of Shandong, China.

References

1 Jaume L, Simó A, Ferran G, M. José S , Georgina A. Woolly apple aphid *Eriosoma lanigerum* Hausmann ecology and its relationship with climatic variables and natural enemies in Mediterranean areas. Bull Entomol Res 2015;105(1):60–69.

2 Zhang Q, Luo WC. Occurrence characteristics of *Eriosoma lanigerum* and its control methods. Entomol Knowledge. 2002;39(5):340–342.

3 Wang XY, Jiang CT, Xu GQ. Potential distribution ofan invasive pest *Eriosoma lanigerum* in China. Chinese Journal of Applied Entomology 2011; 48(2): 379–391.

4 Chen WM, Yu JN, Xu Y, Liu XL, Ru KY. The first occurrence of woolly apple aphid in Xinjiang. Xinjiang Agric Sci2006;4:309.

5 Zhou HX, Yu Y, Tan XM, Chen AD, Feng JG. Biological control of insect pests in apple orchards in China. BiolContr. 2014;68:47–56.

6 Yang QR, Gao ZM, Zheng Y, Wang T. Investigation on the natural enemies of woolly apple aphid. Plant Quarantine. 2005;19(1): 52-52.

7 Kuang RP, Shan F, Tang YZ, Zhong N. Evaluation of natural enemies of woolly apple aphid, *Aphelinus mali* (Hald). Natural Enemies of Insects.1985;11(2): 51-56.

8 Ma M, Guo JY, Tan XM, Sun LN, Zhou HX, Wan FH. *Aphelinus mali*—parasitic wasps with potential to control *Eriosoma lanigerum*. Entomol J East China. 2008;17(1):72-73.

9 Long CD, Wang YP, Tang PZ. Studies on the biological characteristics and utilization of *Aphelinus mal*i Haldeman. Acta Entomol Sinica. 1960;10(1):1-39.

10 Maxwell KB, Susan WK, Robert AW, William AO, David DW, Millicent AC. Cross mating studies among five fruit fly parasitoid populations: potential biological control implications for tephritid pests. BioControl. 2008;53:709–724.

11 Zhang RM, Zhou HX, Guo D, Tao YL, Wan FH. Two putative bridgehead populations of *Aphelinus mali* (Hymenoptera: Aphelinidae) introduced in China. Fla.Entomol. 2014; 97(2):401-405.

12 Zhou HX, ZhangRM, GuoD, TaoYL, WanFH, Wu Q,et al. Analysis of genetic diversity and structure of two clades of *Aphelinus mali* (Hymenoptera: Aphelinidae) in China. Fla. Entomol. 2014;97(2), 699–706.

13 Su M, Tan XM, Yang QM, Zhou HX, Wan FH. Relative efficacy of two clades of *Aphelinus mali* (Hymenoptera: Aphelinidae) for control of woolly apple aphid (Hemiptera: Aphididae) in China. J. Econ.Entomol.2017;110:35–40.

14 Su M, Tan XM, Yang QM, Zhao CM, Wan FH,Zhou HX. Laboratory comparison of two *Aphelinus mali* clades for control of woolly apple aphid from Hebei

Province. China. Bull.Entomol.Res.2018;108(3):400-405.

15 Su M, Zheng XM, Tan XM, Chu D, Zhou HX, Wan FH. Antennae characteristics of *Aphelinus mali* (Haldeman) male and female adults. Shandong Agri Sci.2016;48(7):106-109.

16 Pinto JD, Stouthamer R, Planter GR, Oatman ER. Variation in reproductive compatibility in *Trichogramma* and its taxonomic significance (Hymenoptera: Trichgrammatidae). Ann.Entomol.Soc. Am.1991; 84(1):37-46.

17 Shi ZH, Liu SS, Gebremeskel FB. Comparative study on reproductive compatibility of two geographic populations of *Diadromus collaris* (Gravenhors) (Hym.:Ichneumonidae),a pupal parasitoid of *Plutellaxylostella* (L.) (Lep.:Plutellidae). J Zhejiang Univ.2002;28(2):136-140.

18 Wu QL, Li Q, Gu Y, Shi BC. The complete mitochondrial genome of *Taeniogonalos taihorina* (Bischoff) (Hymenoptera: Trigonalyidae) reveals a novel gene rearrangement pattern in the Hymenoptera. Gene.2014; 543:76–84.

19 Arnett AE, Nicholas JG. Bergmann's rule in the ant lion *Myrmeleon immaculatus* DeGeer (Neuroptera: Myrmeleontidae): geographic variation in body size and heterozygosity. Journal of Biogeography.1999;26,275-283.

20 Sun L, He HM, Xue FS. Geographic Variation of Diapause in Insects. Acta Agriculturae Universitatis Jiangxiensis.2007;29(6):921-927.

21 Tu XY, Xia QW, Chen C, Chen YS, Kuang XJ, Xue FS. Geographic variation in body weight and size of the Asian corn borer, *Ostrinia furnacalis* Guenée (Lepidoptera: Pyralidae). Acta EntomologicaSinica.2011;54(2):143-148.

22 Fairbairn DJ. Allometry for sexual size dimorphism: pattern and process in the coevolution of body size in males and females. Annu Rev Ecol Syst.1997;28:659-687.

23 Deng T, Xue XX. An existing countermeasure of mammals in glacial periods—a new explanation to the Bergmann's Law. Acta Theriologica Sinica. 1997;17(4):259-265.

24 Castro LR, Dowton M. The position of the Hymenoptera with in the Holometabola as inferred from the mitochondrial genome of *Perga condei* (Hymenoptera: Symphyta: Pergidae). Mol Phyl Evol. 2005;34(3):469-479.

25 Cha SY, Yoon HJ, Lee EM, Yoon MH, Hwang JS, Jin BR, et al. The complete nucleotide sequence and gene organization of the mitochondrial genome of the bumblebee, *Bombus ignitus* (Hymenoptera: Apidae). Gene.2007;392(1 -2):206-220.

26 Sun Z, Zhang J, Wang R, Xu YJ, Zhang DQ. Progress of insect mitochondrial genome. J Inspection and Quarantine.2010;20(03):69-73.

27 Wei SJ, Shi M, Chen XX, Sharkey MJ, Van AC, Ye CY,et al. New views on strand asymmetry in insect mitochondrial genomes. PloS ONE. 2010; 5:1767-1780.

28 Du LJ, Wei SJ, Tan XM, Chu XX, Yang QM, Wang JQ, et al. Mitochondrial genome of *Aphelinus mali* (Haldeman) (Hymenoptera: Aphelinidae) with a higher AT percent and the highest AT-skew in Hymenoptera insects. Entomol. News.2020;129(3):267-285.

29 Herrero MI, Fogliata SV, Vera A, Casmuz A, Sosa GD, Castagnaro AP, et al. Biological characterization and mating compatibility of *Helicoverpa gelotopoeon* (D.) (Lepidoptera: Noctuidae) populations from different regions in Argentina. Bull Entomol Res.2018;108(1):108-115.

30 Fogliata SV,Vera A, Gastaminza G, Cuenya MI, Zucchi MI, Willink E, et al. Reproductive isolation between two populations of *Diatraea saccharalis* (F.) (Lepidoptera: Crambidae) from different host plant species and regions in Argentina. Bull Entomol Res.2016;106(5):591-597.

31 Ayala FJ. Genetic differentiation during the speciation process. Evolutionay Biology.1975;8:1–75.

32 Feder JL, Filchak KE. It's about time: the evidence for host plant-mediated selection in the apple maggot fly, *Rhagoletis pomonella*, and its implications for fitness trade-offs in phytophagous insects. Entomol Exp Appl. 1999;91(1): 211–225.

33 Morgan DJW, Reitz SR, Atkinson PW, Trumble JT. The resolution of Californian population of *Liriomyza huidobrensis* and *Liromyza trifolii* (Diptera: Agromyzidae) using PCR. Heredity.2000;85(1): 53–61.

Table 1. Primers used for amplification of the mitochondrial genome

**Fragment No.**	**Gene or region**	**Primer name**	**Sequence (5’-3’)**	**Length(bp)**
F1	*tRNA-Cys-COX3*	RGF1	CCAAWTAAGAAATATAAATATTC	871
		RGR1	CTGGTTTTCATGGTCTTCATG	
F2	*COX3*	RGF2	GTCAATATCAAGCTGCAGC	191
		RGR2	AGAGTATTCTGAAGCTTC	
F3	*COX3-ATP6*	RGF3	GTTCCTATTAAAACATGAAGAC[illegible]	926
		RGR3	CCTTTAGGGACACCAGGAG	
F4	*ATP6*	RGF4	GACCAGCAATCATATTTGCAG	486
		RGR4	CTGTTTTTGATCCTTCTAC	
F5	*ATP6-COX1*	RGF5	ATAACGAGAAGGAATAAATC	1876
		RGR5	GAATGGATGTAGATACTCG	
F6	*COX1*	RGF6	CCATTTATTGAAGCAAGTCATC	616
		RGR6	CTGGGACAGGTTGAACTG	
F7	*COX1-NAD5*	RGF7	TTGAACCTATAATTGAAG	1346
		RGR7	GTCAAGATATTCGTTTTATAGG	
F8	*NAD5*	RGF8	AAGGTATTCCACATAATG	710
		RGR8	TGGGTGGGATGGTTTAGG	
F9	*NAD5-NAD4*	RGF9	TATTACATCTCCAATTCG	1073
		RGR9	GTTTGTTTACGTCAGTATG	
F10	*NAD4*	RGF10	GCTGAAGAACATAAACCATG	331
		RGR10	GACTTCCTAAAGCTCATG	
F11	*NAD4-CYTB*	RGF11	CGAACAACACCATAACCC	2239
		RGR11	GAATAAAATCGATTTAGTGTAGC	
F12	*CYTB*	RGF12	TGAGGAGCTACTGTTATTAC	396
		RGR12	TCATTCAGGTTGAATATG	
F13	*CYTB-NAD1*	RGF13	CCATATATTTTAAGAGACCC	1475
		RGR13	GATTTATAATAATTAGGTTTCCA[illegible]	
F14	*NAD1*	RGF14	CCTTTCATATATTCTGTG	397

		RGR14	GAAATAATTTACCATTTTATTCTC	
F15	*NAD1-RRNL*	RGF15	GAGCAATACTCCGAAGAC	697
		RGR15	GTTTGCGACCTCGATGTTG	
F16	*RRNL*	RGF16	CTCACGCCGATCTAAACTC	1051
		RGR16	CCTTTTGTATCAGGGTTA	
F17	*RRNL-RRNS*	RGF17	TTAATCATTGAGCAGATC	871
		RGR17	GTACAAATTGCCCGTCGC	
F18	*RRNS*	RGF18	ACTATATTCCTAACTTCT	461
		RGR18	AACTAGGATTAGATACC	
F19	*RRNS-tRNA-Asn*	RGF19	CAATTACAAAACAAGTTCCTC	904
		RGR19	CTTAATTGGAAAAATAATTTC	

Table2 Morphological measurements of two clades of *Aphelinus mali* in China.

Character length		Females (Mean ± SD)		Males (Mean ± SD)	
		Shandong	Liaoning	Shandong	Liaoning
Whole body		810.93 ± 16.03b	870.29 ± 12.18a	679.34 ± 12.34d	758.38 ± 15.95c
Abdomen		384.12 ± 9.75b	424.43 ± 7.87a	292.96 ± 7.53d	339.94 ± 8.04c
Forewing		682.40 ± 11.09c	770.86 ± 9.39a	609.84 ± 8.42d	735.96 ± 8.35b
Hind wing		303.46 ± 4.99c	336.84 ± 4.14a	277.70 ± 4.02d	321.52 ± 3.51b
Anterior leg	Femur	176.70 ± 3.37c	195.25 ± 2.52a	167.46 ± 2.14d	188.30 ± 2.24b
	Tibia	147.32 ± 2.67b	163.43 ± 2.29a	138.67 ± 2.20c	159.98 ± 2.36a
	Tarsus	158.73 ± 2.87b	168.58 ± 2.87a	150.56 ± 2.18c	166.04 ± 2.48a
	Pre-tarsus	19.36 ± 0.41b	24.14 ± 1.36a	19.54 ± 0.46b	23.31 ± 0.38a
	Spur	26.23 ± 0.96bc	27.56 ± 0.56b	25.72 ± 0.71c	29.17 ± 0.76a
	Total leg	528.32 ± 8.42b	551.39 ± 6.96a	476.22 ± 6.33c	543.33 ± 9.01a
Middle leg	Femora	214.75 ± 3.14c	238.31 ± 3.11a	200.52 ± 3.22d	228.08 ± 2.85b
	Tibia	249.91 ± 4.53c	276.08 ± 3.51a	229.60 ± 3.50d	265.51 ± 3.05b
	Tarsus	202.98 ± 3.74b	215.30 ± 3.24a	191.74 ± 2.87c	210.43 ± 3.03a
	Pre-tarsus	19.36 ± 0.41b	23.26 ± 0.29a	19.87 ± 0.42b	22.82 ± 0.29a
	Spur	66.91 ± 1.67b	74.05 ± 1.15a	66.27 ± 1.40b	72.19 ± 2.87a
	Total leg	753.51 ±12.23a	752.95 ± 9.44a	641.73 ± 9.09c	728.55 ± 10.62b
Hind leg	Femora	253.72 ± 4.56a	256.15 ± 3.75a	223.63 ± 3.44c	239.85±2.87b
	Tibia	259.67±4.06c	288.79±4.02a	231.29 ± 3.68d	274.67 ± 3.75b
	Tarsus	226.86 ± 4.23b	242.70 ± 3.72a	211.99 ± 3.41c	233.09 ± 3.30b
	Pretarsus	19.79 ± 0.42b	23.30 ± 0.34a	19.64 ± 0.44b	23.24 ± 0.32a
	Spur	32.20 ± 1.16c	36.54 ± 0.73a	28.60 ± 0.92d	34.16 ± 0.68b
	Total leg	792.33 ± 12.32a	810.94 ± 11.57a	686.55 ± 9.61c	768.52 ± 12.10b
Antenna	Funicle1	16.52 ± 0.44b	19.03 ± 0.31a	16.27 ± 0.43b	18.96 ± 0.32a
	Funicle2	18.04 ± 0.30b	19.33 ± 0.30a	16.44 ± 0.29c	18.94 ± 0.30a
	Funicle3	33.12 ± 0.44c	36.57 ± 0.54b	46.83 ± 0.76a	45.18 ± 1.52a
	Club	91.13 ± 1.37d	106.76 ± 1.03b	100.10 ± 2.19c	113.90 ± 1.72a
	Total antenna	158.81 ± 2.18c	181.68 ± 1.68b	179.62 ± 2.92b	212.50 ± 2.55a

Data are mean ± SD (μ m), and different letters in the same row indicate significant differences.

Table 3 Comparisons of the mitochondrial genome between two clades of *Aphelinus mali* in China

Gene	Clade	Strand	Length(bp）	Anti/start codon	Stop codon	Intergenic nucleotides
COX3	Shandong	-	732	ATG	TAA	39
	Liaoning	-	732	ATG	TAA	2
ATP6	Shandong	-	651	ATT	TAA	3
	Liaoning	-	651	ATT	TAG	-10
ATP8	Shandong	-	156	ATT	TAA	-10
	Liaoning	-	162	ATT	TAA	24
TRND	Shandong	-	68	GTC		24
	Liaoning	-	71	GTC		10
TRNK	Shandong	+	70	TTT		9
	Liaoning	+	74	TTT		2
COX2	Shandong	-	660	ATT	TAA	2
	Liaoning	-	660	ATT	TAA	0
TRNL2	Shandong	-	68	TAA		0
	Liaoning	-	67	TAA		14
COX1	Shandong	-	1512	ATT	TAA	-18
	Liaoning	-	1509	ATT	TAA	-18
TRNE	Shandong	+	67	TTC		4
	Liaoning	+	67	TTC		-1
TRNF	Shandong	-	70	GAA		-1
	Liaoning	-	67	GAA		0
NAD5	Shandong	-	1479	ATT	TAA	11
	Liaoning	-	1563	ATT	T	12
TRNH	Shandong	-	68	GTG		0
	Liaoning	-	68	GTG		0
NAD4	Shandong	-	1335	ATG	T	-7
	Liaoning	-	1308	ATG	T	-7
NAD4L	Shandong	-	273	ATA	TAA	-7
	Liaoning	-	267	ATA	TAA	-7
TRNT	Shandong	+	66	TGT		0
	Liaoning	+	66	TGT		
TRNP	Shandong	-	65	TGG		2
	Liaoning	-	64	TGG		
NAD6	Shandong	+	519	ATA	T	15
	Liaoning	+	513	ATA	T	1

Gene	Clade	Strand	Length(bp）	Anti/start codon	Stop codon	Intergenic nucleotides
COB	Shandong	+	1083	ATA	TAA	1
	Liaoning	+	1083	ATA	TAA	27
TRNS2	Shandong	+	65	TGA		27
	Liaoning	+	65	TGA		73
TRNS2	Shandong	+	65	TGA		77
	Liaoning	+	70	TGA		77
TRNS2	Shandong	+	65	GGA		78
	Liaoning	+	65	GGA		77
TRNS2	Shandong	+	65	TGA		149
	Liaoning	+	65	TGA		149
NAD1	Shandong	-	891	ATA	TAA	0
	Liaoning	-	891	ATA	TAA	0
TRNL1	Shandong	-	66	TAG		0
	Liaoning	-	66	TAG		0
RRNL	Shandong	-	1359			0
	Liaoning	-	1360			0
TRNA	Shandong	-	70	TGC		0
	Liaoning	-	67	TGC		0
RRNS	Shandong	-	769			0
	Liaoning	-	771			0
TRNV	Shandong	-	56	TAC		323
	Liaoning	-	56	TAC		20
NAD2(P)	Shandong	-	240	ATT	ATT	40
	Liaoning	-	213	ATT	TGA	
TRNN	Shandong	+	66	GTT		0
	Liaoning	+	66	GTT		
TRNC	Shandong	-	64	GCA		
	Liaoning	-	64	GCA		44
TRNR	Shandong	-	59	TCG		
	Liaoning	-	59	TCG		2
NAD3	Shandong	-	312	ATA	TAA	
	Liaoning	-	312	ATA	TAA	-3
TRNG	Shandong	-	65	TCC		
	Liaoning	-	65	TCC		41
//	Shandong		998			
	Liaoning		972			

Table4 The base content, AT skewness and GC skewness of the two *Aphelinus mali* clades compared to other Hymenoptera

Species	T%	C%	A%	G%	A+T%	ATskew	GCskew
Aphelinus mali SC	36.69	9.40	47.30	6.61	84.44	0.1291	-0.1619
Aphelinus mali LC	36.69	9.40	47.30	6.61	83.99	0.1305	-0.1328
Taeniogonalos taihorina	43.51	6.77	41.09	8.63	84.60	-0.0286	0.1208
Spathius agrili	45.12	6.55	38.88	9.45	84.00	-0.0743	0.1813
Cotesia vestalis	47.46	5.78	39.70	7.06	87.16	-0.0890	0.0997
Enicospilus sp.	43.44	8.73	41.45	6.08	85.19	-0.0198	-0.1789
Abispa ephippium	39.50	14.60	39.10	6.70	78.60	-0.0051	-0.3709
Bombus hypocrita	42.56	9.68	42.77	4.98	85.33	0.0025	-0.0275
Perga condei	33.80	14.60	42.80	8.80	76.60	0.1175	-0.2479

SC: Shandong clade, LC: Liaoning clade

Table 5 The base composition of mitochondrial genomic coding gene and rRNA gene of two clades of *Aphelinus mali* in China

Gene	Clades	T％	C％	A％	G％	A＋T％	AT skew	GC skew
COX3	Shandong	30.13	12.05	49.23	8.59	79.36	0.2454	-0.1676
	Liaoning	29.87	12.44	49.10	8.59	78.97	0.2435	-0.1831
ATP6	Shandong	30.83	8.85	50.88	9.44	81.71	0.2454	-0.0323
	Liaoning	31.56	9.29	50.15	9.00	81.71	0.2282	-0.0159
ATP8	Shandong	42.42	4.85	49.09	3.64	91.52	0.0729	-0.1427
	Liaoning	41.82	4.85	49.09	4.24	90.91	0.0800	-0.0671
COX2	Shandong	34.51	11.01	46.26	8.22	80.76	0.1455	-0.1450
	Liaoning	34.80	11.01	45.81	8.37	80.62	0.1366	-0.1362
COX1	Shandong	31.66	12.81	45.75	9.78	77.41	0.1820	-0.1341
	Liaoning	30.82	13.32	46.27	9.59	77.09	0.2004	-0.1628
NAD5	Shandong	33.21	.72	52.21	5.85	85.42	0.2224	-0.1968
	Liaoning	33.13	8.49	52.63	5.74	85.77	0.2274	-0.1933
NAD4	Shandong	34.28	8.79	50.67	6.26	84.95	0.1929	-0.1681
	Liaoning	32.11	11.60	47.01	9.28	79.12	0.1883	-0.1111
NAD4L	Shandong	34.01	8.42	54.55	3.03	88.55	0.2320	-0.4707
	Liaoning	35.35	7.74	54.88	2.02	90.23	0.2164	-0.5860
NAD6	Shandong	45.82	6.00	44.91	3.27	90.73	-0.0100	-0.2945
	Liaoning	44.91	6.79	45.06	3.24	89.97	0.0017	-0.3539
COB	Shandong	43.73	10.93	35.29	10.04	79.02	-0.1068	-0.0424
	Liaoning	42.81	11.58	35.53	10.09	78.33	-0.0929	-0.0688
NAD1	Shandong	33.76	11.75	47.44	7.05	81.20	0.1685	-0.2500
	Liaoning	33.44	12.39	47.22	6.94	80.66	0.1708	-0.2818
NAD3	Shandong	33.33	9.32	52.26	5.08	85.59	0.2212	-0.2942
	Liaoning	33.33	9.60	51.41	5.65	84.75	0.1661	-0.2590
RRNL	Shandong	41.20	7.46	46.76	4.57	87.97	0.0632	-0.2352
	Liaoning	41.08	7.55	46.95	4.42	88.03	0.0667	-0.2615
RRNS	Shandong	42.39	7.67	46.42	3.51	88.82	0.0454	-0.3721
	Liaoning	42.15	7.65	46.82	3.37	88.98	0.0525	-0.3884
NAD2	Shandong	35.00	5.00	55.00	5.00	90.00	0.2222	0
	Liaoning	35.92	4.92	54.93	4.23	90.85	0.2092	-0.0754

Table 6 Results of crosses between the two *Aphelinus mali* clades, in terms of the number of pupae, progeny produced, emergence rate, female ratio and relative compatibility of hybridization.

	# pupae	# adult progeny	% emergence	Sex ratio (%F)	relative compatibility
L♂×S♀（n=18）	5.28 ± 2.93a	4.67 ± 3.03ab	85.09± 16.75a	0.57 ± 0.28a	1.188
S♂×L♀（n=15）	4.86 ± 4.28a	3.78 ± 3.62a	77.94 ± 28.87a	0.53 ± 0.32a	0.964
S♂×S♀（n=15）	7.00 ± 4.95a	6.37 ± 1.69ab	91.00 ± 13.98a	0.48 ± 0.33a	——
L♂×L♀（n=18）	8.67 ± 2.89a	7.29 ± 2.39b	84.08± 30.29a	0.55 ± 0.34a	——
	df=3 F=1.147<br>P=0.343	df=3 F=2.058<br>P=0.123	df=3 F=1.076<br>P=0.372	df=3 F=0.133<br>P=0.940	——

Data in the same column are means ± SD, different letters indicate significant differences (*P*<0.05). L: Liaoning clade. S: Shandong clade

Table 7 The number of pupae, progeny produced, emergence rate, sex ratio (as %F)and relative compatibility of backcross.

	# pupae	# progeny	% emergence	Sexratio (% F)	relative compatibility
（S♀×L♂）♂backcrossS♀ （n=5）	3.80 ± 2.95a	3.40 ± 3.88b	77.50 ± 43.66a	0.47 ± 0.35a	0.980
（S♀×L♂）♀backcrossL♂ （n=3）	4.33 ± 1.53a	4.00 ± 1.00ab	94.40 ± 9.62a	0.59 ± 0.08a	1.230
（S♂×L♀）♂backcrossL♀ （n=3）	3.33 ± 1.53a	3.00 ± 2.00b	83.30 ± 28.87a	0.76 ± 0.21a	1.382
（S♂×L♀）♀backcrossS♂ （n=3）	6.67 ± 2.08a	6.33 ± 2.52ab	93.30 ± 11.55a	0.61 ± 0.10a	1.110
S♂×S♀ （n=15）	7.00 ± 4.95a	6.37 ± 1.69ab	91.00 ± 13.98a	0.48 ± 0.33a	——
L♂×L♀ （n=18）	8.67 ± 2.89a	7.29 ± 2.39a	84.08± 30.29a	0.55 ± 0.34a	——
	df=5 F=1.488 P=0.249	df=5 F=1.829 P=0.164	df=5 F=0.386 P=0.851	df=5 F=0.615 P=0.690	——

Data in the same column are mean ± SD, different letters indicate significant differences (P<0.05). L: Liaoning clade; S: Shandong clade.

Figure Click here to access/download;Figure;11-14 figure.docx

Fig. 1. Sampling sites and haplotype frequencies in the 16 populations of *A. mali*

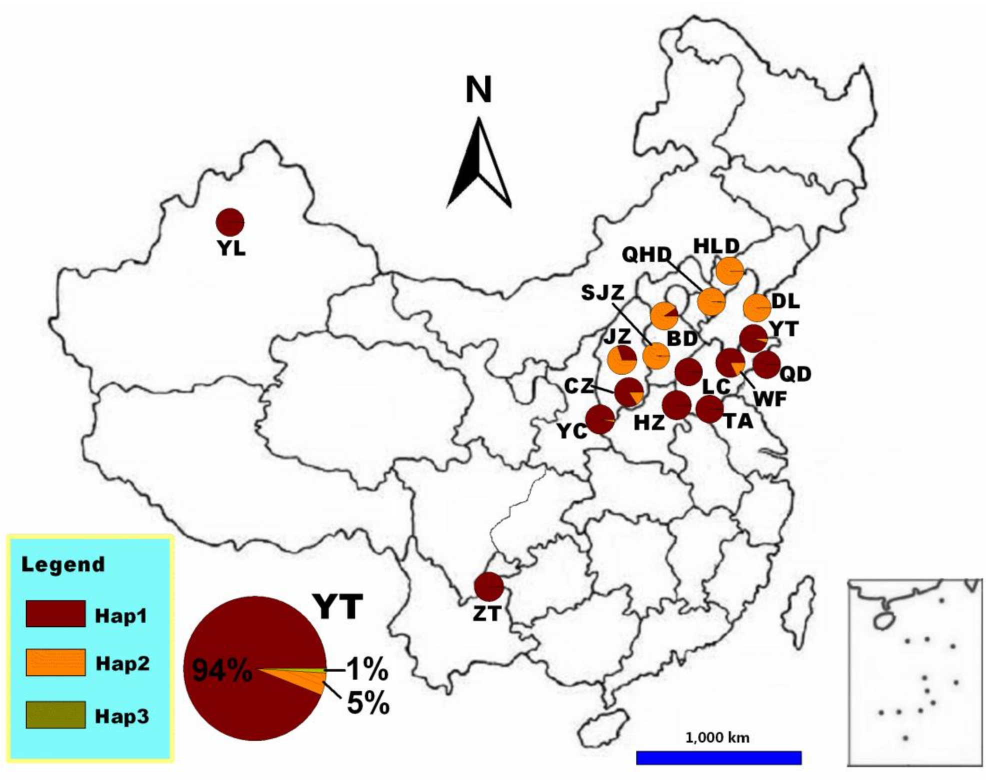

Fig. 2. The images of male and female from two clades of *A.mali*

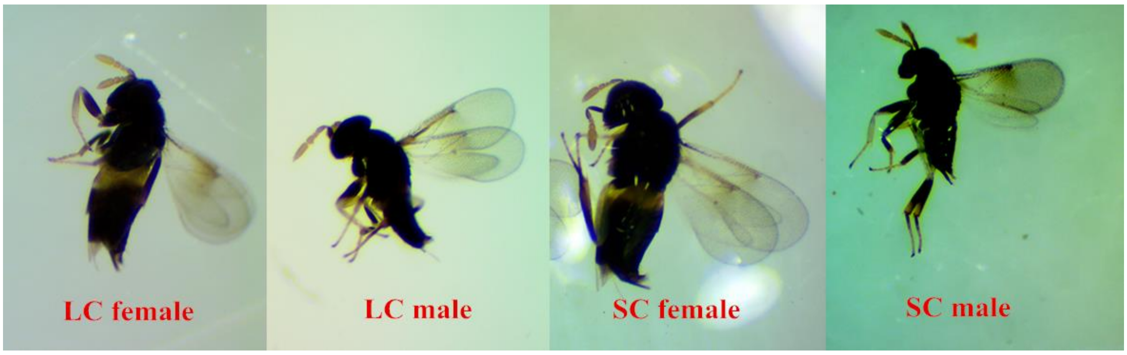

Note: LC: Liaoning Clade; SD:Shandong Clade

Fig. 3. The linear arrangement of mitochondrial genomes in the sequences of two clades

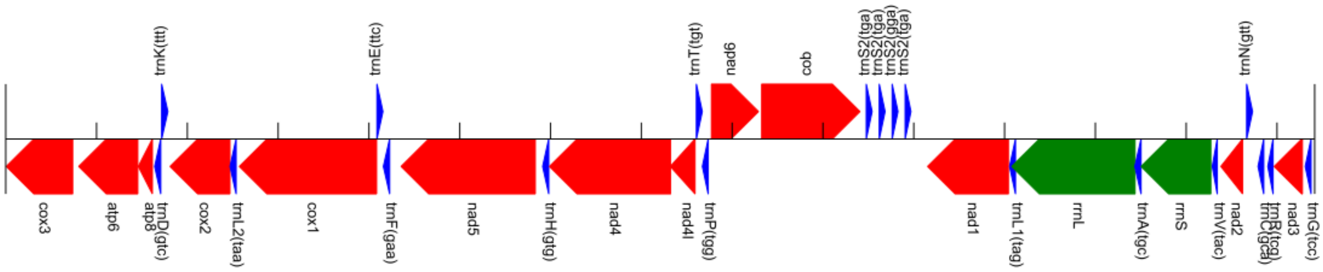

Shandong clade

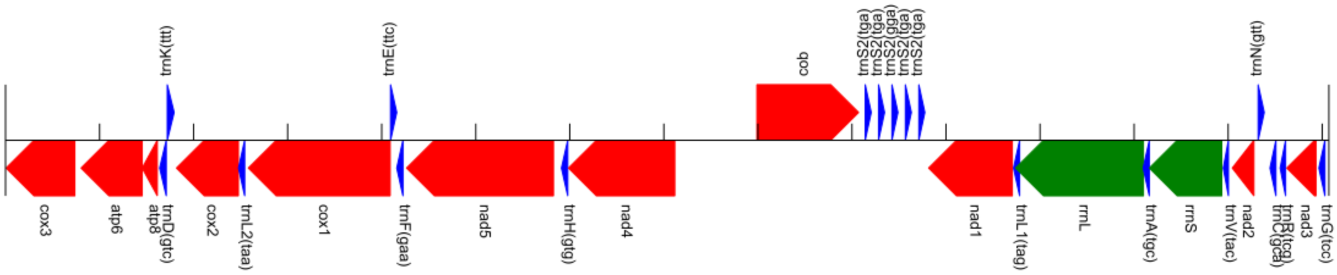

Liaoning clade

Click here to access/download
Supporting Information
11-14 table.docx

Differences in morphology, mitochondrial genomes, and reproductive compatibility between two clades of parasitic wasps *Aphelinus mali* (~~Hemiptera~~Hymenoptera: Aphelindae~~ididae~~) in China

Min Su~~Min~~[a,b], Lingjun Du ~~Lingjun~~[a], Yasir Ali Muhammad ~~Yasir Ali~~[a], Jianing Yu-~~Jianing~~[a], Mengyu Chi ~~Mengyu~~[a], Ziwen Teng ~~Ziwen~~[a], Yinjun Fan ~~Yinjun~~[a], Xiumei Tan-~~Xiumei~~[a], Hongxu Zhou~~Hongxu~~[a,*]

[a]College of Botanical Medicine, Qingdao Agricultural University, Key Laboratory of Integrated Plant Disease and Pest Control, China-Australia Joint Institute of Agricultural and Environmental Health, Qingdao 266109, China (18765903010@163.com; 1163355473@qq.com; m.yasirali4663@gmail.com; 2354846762@qq.com; 1538848218@qq.com; tzwbat@126.com; fanyinjun89@126.com; xmtanly@163.com; hxzhouqd@sina.cn)

[b]Agricultural and rural Bureau of Zhangqiu District, Jinan 250200, China (18765903010@163.com)

* Corresponding author(hxzhouqd@sina.cn)

Min Su ~~Min~~ helped write the article; Lingjun Du ~~Lingjun~~contributed the work on the mitochondrial genomes; Jianing Yu ~~Jianing~~ contributed to the part on reproductive compatibility; Mengyu Chi ~~Mengyu~~ contributed to the part on morphological characteristics; Yasir Ali Muhammad, Ziwen Teng, Yinjun Fan, Xiumei Tan and Hongxu Zhou checked the manuscript.~~.~~

Abstract

Aphelinus mali (Haldeman) (Hymenoptera: Aphelinidae) in China is comprised of two clades (termed, the Shandong and Liaoning clades). In order to clarify the genetic relationship, between these two clades, we compared and analyzed the morphological characteristics and the mitochondrial genome of each, and performed a hybridization experiment. ~~between them, we compared and analyzed the morphological characteristics and the mitochondrial genome of each, and ran a hybridization experiment between these clades.~~Morphological results showed that both males and females of the Liaoning clade were larger than in the Shandong clade, in terms of whole body, abdominal, wing and antennal lengths, but there were no significant differences between clades for total length of the middle or hind leg of females. The length of the mitochondrial genome of the Shandong clade was14415 bp and, for the Liaoning clade, it was 14804 bp. Each contained 31 genes, including 13 protein-encoded genes, 16 tRNA genes, and 2 rRNA genes. The highest AT level~~s~~ among the 13 protein-coding genes for the two clades were ~~for~~ the same gene (*ATP8)* (Shandong clade, 91.52%; Liaoning clade, 90.91%). By hybridization and backcrossing, we found there was no cross incompatibility between these two clades of *A. mali*. Our results indicate that the historic geographical isolation between these

clades has not yet caused reproductive isolation of these populations, and they belong to the same species.

Key words *Aphelinus mali*, hybridization, morphological characteristics, mitochondrial genome

Introduction

Woolly apple aphid, *Eriosoma lanigerum* (Hausmann) (Hymenoptera: Aphididae), is an important quarantine pest of apples in many parts of the world[1] (Jaume et al. 2015). In recent years, the damage caused by this aphid has become a serious problem in China, surveys in Rizhao, Shandong Province from 2000 to 2002, found WAA in about 8000 hectares of orchard, with 10–20% of the trees being infested, causing an annual loss of 5 × 106 kg of apples (Wang et al., 2011), and the pest's range has continued to increase[2-4] (Zhang and Luo 2002, Chen et al.2006). The endoparasitoid *Aphelinus mali* (Haldeman) (Hymenoptera: Aphelinidae) is the dominant natural enemy of *E. lanigerum* in China[5] (Zhou et al. 20214)and is considered the most effective of the aphid's bio biocontrol agents[6,7](Yang et al. 2005, Kuang et al. 1985) . *Aphelinus A. mali* has been introduced into 51 countries and has established in 42 of them[8] (Ma et al. 2008).

AphelinusA . mali was introduced into China twice: the first introduction was from

Japan into Dalian and Lvshun, Liaoning Province (122°31'E, 39.20°N) in 1942, while the second was from the former Soviet Union in 1950 into Qingdao, Shandong Province (116°41'E, 39°91'N) [9]~~(Long et al. 1960)~~. High intra-specific variability is expected when a species has a large geographic distribution, as does *A. mali*[10] ~~(Maxwell et al. 2008)~~. Based on the study of the mitochondrial COI gene of this parasitoid, ~~we found that~~*A. mali* in China is comprised of two regional clades (named the Shandong and Liaoning clades) [11,12](Fig.1)~~, and that t~~. The *A. mali* population in Qingdao and Tai'anin Shandong province and the population in Dalian and Huludao in Liaoning province ~~(Zhang et al. 2014, Zhou et al. 2014)~~differ in their biological characteristics and control potential[13,14] ~~(Su et al. 2017, Su et al. 2018)~~.

The study of the genetic relationship between these two clades can help to assess the ecological adaptability of each clade and determine whether they have undergone intra-specific differentiation. Such information can provide new ideas and methods for the introduction, reproduction, and field release of *A.mali* as a biological control agent against *E. lanigerum*. The morphological characteristics and mitochondrial genome were compared to clarify the genetic relationships between two clades of *A.mali*. ~~We~~ The experimenters also performed a~~ran a~~ hybridization experiment to see if any reproduction isolation has developed between these clades.

Materials and Methods

Morphological comparison of clades. To obtain parasitoids for morphological comparisons, ~~wecollected~~*E. lanigerum* aphids were collected from apple orchards in Tai'an (117°13E, 36°19N), Shandong province and Dalian (121°52E, 38°95N),

Liaoning province, in mid-August, 2014. Parasitized aphids which black and mummified were noted and placed in Ppetri dishes and held atca25at 25°C for parasitoid emergence. Adults of A. mali were removed dailyevery day, placed in 100% ethanol, and conservedstored at 20°C. Male and female *A. mali* were separated based on the larger body size of females, antennal shape (the third section of the female's antenna at the funicular joint is square, while that of the male is rectangular)[15] (Su et al. 2016), and the abdominal shape (females have a short, thick abdomen, while the male's abdomen is slender)[9] (Long et al. 1960).

Images of body regions or parts were taken with the universal video imaging system (LY-WN-HPCCD (10),Chengdu Li Yang Precision Mechanical and Electrical Co., Ltd., China). This systemwas used to capture photographs of morphological parts, and wWe then measured the length of the body and abdomen, the length and width of front and hind wing, the length of the whole leg, the tibia, tarsae, anterior tarsal segment, and calcar (spine at the tip of the tibia) of the foreanterior, middle,, and hind legs, the length and width of the rod section of the antennal, and the first, the second, and the third antennal segments. One hundred adults of *A. mali* (50M and 50F) from each sample area were measured.

Mitochondrial genomic genome sequencing. For mitochondrial sequencing, – wecollected aphids parasitized by *A. mali* were collected from one abandoned apple orchard in Tai'an, Shandong Province and another in Huludao (120°87E, 40°77N), Liaoning province. At each site, the five-point sampling method was used, sampling ten trees at each of the five points. On each of these 50 trees per location, five

branches bearing blackened *E. lanigerum* aphids that were parasitized by *A. mali* were collected and put into a 25°C incubator in the laboratory to await for*A. mali* emergence. One hundred adults of *A. mali* were obtained from each site and placed individually in 1.5 ml centrifuge tubes containing absolute ethanol. and sent to Wuhan Transduction Bio Co., LTD for sequencing.

Primers designed to match generally conserved regions of target mtDNA were used to amplify short fragments from *COX3, ATP6, COX1, NAD5, NAD4, CYTB, NAD1, RRNL, RRNS* (Table 1). Specific primers were designed based on these conserved regions sequences and used to amplify the remained mtDNA sequence in several PCR reactions. Primers were designed to produce amplicons with overlaps of about 100 bp. The PCR reaction was performed using the LA Taq polymerase. The PCR conditions were as follows: initial denaturation 94°C for 2 min, then 35 cycles of denaturation at 94°C for 30s, annealing at 55°C for 30s, and extension at 72°C for 1 min/kb, followed by the final extension at 72°C for 10 min. The total volume for PCR and LA-PCR was 50 µL, of which 0.5 µL Takara LATaq (5 U/µL), 5 µL10×LATaq Buffer II (Mg^{2+}), 8 µLdNTP Mixture (2.5 mM), 60 ng Template, and the total volume was then made up with distilled water. The final concentration of the forward and reverse primers was 0.2~1.0 µM, and that of $MgCl_2$ was 2.0 mM. The PCR products were sequenced directly, or if needed first cloned into a pMD18-T vector (Takara, JAP) and then sequenced, by the dideoxynucleotide procedure, using an ABI 3730 automatic sequencer (Sanger sequencing) using the same set of primers. All obtained fragments were quality-proofed (electropherogram) and BLASTed to confirm that the amplicon

Formatted: Level 2

Formatted: Font: Italic

Formatted: Superscript

Formatted: Subscript

is the actual target sequence. Whenever the quality was sub-optimal, sequencing was repeated. All obtained fragments were BLASTed to confirm that the amplicon is the target sequence. Mitogenome was assembled stepwise with the help of DNAstar v7.1 program, making sure that the overlaps were identical, and that no NUMTS were incorporated into the sequence.For DNA extraction, we used the Takara Genomic extraction Kit, specific PrimerLA Taq Polymerase, $MgCl_2$ solution and pMD18-T Vector.For sequencing we used the following procedure. First, primers were designed for the general conserved region to amplify the short regions of the 16s, 12s, cox1, atp6, cox3, nad4, cytb, and nad1 genes, and then specific primers were designed to amplify the remaining sequences according to the amplified conserved sequences. The resulting long fragments were then amplified using LA Taq polymerase, and the PCR program was run through 35 cycles (specific process: 94°C for 30 s, annealed at 50°C for 30s, extension at 72°C for 1 min per 1kb.). The PCR product was then cloned into a PMD-18T (Takara, JAP) vector or sequenced directly with PCR product in ABI3730 automatic sequencer. The sequencing results were then spliced and assembled by DNASTAR software, and the complete mitochondrial sequence was obtained by manual analysis and adjustment. For amplification of the product, we then added 0.5μLTaKaRa LA Taq (5U/μL), 5μL 10×LA TaqBufferII (Mg^{2+}Plus), 8 μL dNTP Mixture (2.5 mM each), 60 ng Template, 0.2–1.0μm (final conc.), Primer F and Primer R, 0.2–1.0μm (final conc.), adding ddH_2O to make up the solution to 50 μL.

Formatted: Pattern: Clear (White)

Cross mating experiment. For the cross-mating experiment, we collected *A.mali* as parasitized aphids on apple trees (as described below) were collected from Qingdao

(120°39E, 36°31N) to represent the Shandong clade and from Qinhuangdao (119°48E, 39°83N) from the Liaoning clade.

The experimenters~~We~~ choose an apple orchard with less management and protected the apple trees that had colonies of *E. lanigerum* with screen cages (1m×1m×2m). Thereafter, ~~ wepick out~~the parasitized black aphids were eliminated daily until it ~~doesn't~~ didn't appear, ~~make~~ made sure there were no *A. mali* in the *E. lanigerum* colony, and then kept it in its natural state in the apple orchard, waiting for parasitized by the *A.mali* in our experiment.~~.~~

Formatted: Font: Italic

Crosses and reciprocal crosses between the two populations (one per clade) were made using within clade crosses in each population as the control group (Liaoning♀×Shandong♂, Liaoning♂×Shandong♀); all crosses were repeated 15-20 times. The *A. mali* were held in the Petri dishes (13.5 cm diameter, had cotton ball with 10% honey water) at 25℃, 70% RH, and a 16:8 h L:D photoperiod. For each cross, we recorded the number of hosts attacked by each mated, crossed female and the number of ~~her~~ offspring, and these offspring's sex ratio (as % female).

The F1 generation of each hybrid cross were backcrossed with the parents of each population, conducting a "within hybrid (F1) population" cross as the control group, with 3-5 replicates per type of crossing. We recorded the number of ~~hosts attackedandthe number of~~ parasitoid offspring for each crossed female, for all crossing combinations, as well as the mean sex ratio (MSR)~~sex ratio~~ of the resulting backcrossed progeny (as % female).

~~In each cross, the numbers of male and female progeny were counted and the mean~~

Formatted: Left

~~sex ratio (MSR) for that cross was calculated as the percentage of female progeny.~~

The relative compatibility of an inter-group crossing (A x B) was expressed as[16,17]~~(Pinto et al. 1991, Shi et al. 2002)~~:

$$\frac{\mathrm{MSR}(\mathrm{A}\text{♀} \times \mathrm{B}\text{♂})}{\mathrm{MSR}(\mathrm{A}\text{♀} \times \mathrm{A}\text{♂})} \times 100\%$$

Statistical analysis. Morphological measurements as well as the hybridization and backcross rates are presented as means ± standard deviations (SD), calculated using Statistical Product and Service Solutions (SPSS) 19.0. Significant differences were determined using one-way analysis of variance (ANOVA) corrected by SPSS 19.0. The independent samples t-test was used to analyze the data on parthenogenesis between two clades, using SPSS 19.0.

For genomic analysis, we used MITOS Webserver (http:// mitos. bioinf. uni-leipzig. de/index. py) to annotate and analyze the mitochondrial genome sequence and obtain the linear alignment of the mitochondrial genome sequence. The AT-skew and GC-skew were calculated using DNASTAR as follows: AT-skew = (A% -T%)/ (A% +T%) and GC-skew = (G%-C %)/ (G%+C%).

Results

Comparison of morphological characters. Measurements of body dimensions (Table ~~1~~2) showed that both males and females from the Liaoning clade were bigger than those of the Shandong clade (Fig.2),in terms of the whole body, abdominal, wing, and antennal segment lengths. But the total length of the middle and hind legs showed no significant differences in females between two clades.

Mitochondrial genome sequencing and analysis. The mitochondrial genome had

14415 bp for the Shandong clade and 14804 bp for Liaoning. Both clades contained 31 genes, including 13 protein-encoded genes, 16 tRNA genes, and 2 rRNA genes (Table ~~2~~3, Fig.~~1~~3). The unmeasured area was in the A+T-rich region, which usually contains six tRNA genes (*TRNY, TRNM, TRNI, TRNQ, TRNW,* and *TRNS1*) in such genomes, but which were absent in *A. mali*. Genomes of both clades had a higher A+T content and AT skew than other Hymenoptera, with rearrangements of the tRNA and protein-coding genes. The A+T content in the measured region of the Shandong clade was 84.44%, the AT skew was 0.1291, and the GC skew was -0.1619. The A+T content in the measured region of Liaoning clade was 8~~4~~3.99~~0~~%, the AT skew was 0.1305, and the GC skew was -0.1328 (Table ~~3~~4).For comparison, other Hymenoptera have the A+T content range from 76-86%[18]~~(Wu et al. 2014)~~.

Among the 13 protein-coding genes, the gene with the highest AT content was the *ATP8* gene, in both clades, with an AT content of 91.52% for the Shandong clade and 90.91% for the Liaoning clade. Also in both clades, the protein-coding gene with the lowest AT content was the cox1 gene, being 77.41% for the Shandong clade and 77.09~~1~~% for the Liaoning clade (Table ~~4~~5).

There were two ribosomal RNA genes, *RRNL* and *RRNS*. The lengths of the *RRNL* gene were 1359bp (in the Shandong clade) versus 1360bp (Liaoning clade), and for these ribosomal genes, the AT content was ~~88~~87.97~~0~~% for the Shandong clade and 88.03% for the Liaoning clade,~~.~~and the *RRNL* gene occurred in an area between the *TRNL1* gene and the *TRNA* gene~~)~~. The *RRNS* gene had 769bp (Shandong clade) compared to 771bp for the Liaoning clade, and the AT content of the *RRNS* gene was

88.82% in the Shandong clade and 8~~9.0~~8.98% in the Liaoning clade, and this gene occurred in an area between the *TRNA* gene and the *TRNV* gene.

The length of the tRNA genes of the two clades ranged from 59bp to 75bp in the mitochondrial genome. Three of the 16 tRNA genes, *TRNS2*, *TRNR* and *TRNV*, could not form the normal clover-shaped secondary structure, unlike the other 13 tRNA genes which could do so in both clades.

Hybridization experiment. In both clades, unmated ~~females of~~*A.mali* ~~t~~females~~hat had not mated~~ could parasitize *E. lanigerum* aphids and all offsprings were males, indicating neither clade was parthenogenetic.

Crosses between the two clades of *A.mali* led to the production of fertile offsprings (F1 generation). There were no significant differences in emergence rates or sex ratio (% F) among progeny between clades (Table ~~5~~6).The relative compatibility of the crosses was greater than 0.75 in both crossing directions (L♂×S♀: 1.188, S♂×L♀: 0.964).

In backcrosses, there were no significant differences in the emergence rates or sex ratio (% F) of progeny from back crosses between patental clades and the hybrids from crossings of two clades. The relative compatibility values of the two clades were all greater than 0.75 (Table ~~6~~7).

Discussion

We found differences in some morphological characteristics between the two clades of *A.mali* present in China. Because there were 6 tRNAs that were not detected in

both clades, ~~However,~~ the mitochondrial genomes general structure of the two clades could not be determined and compared ~~because there were 6 tRNAs that were not detected in both clades~~. Also, the secondary structures of the *TRNS2*, *TRNR*, *TRNV* genes were incomplete, and there were two tRNA mismatches between clades. Crosses between the two clades produced fertile F1 females, showing that there were no reproductive barriers between these two geographical populations, and that they have not evolved into two species.

Within species, the body size, developmental duration, and diapause responses of insects frequently vary in different geographical regions[19-21] ~~(Amy and Nicholas 1999, Sun et al. 2007, Tu et al. 2011)~~. The body size of animals often changes along gradients of latitude or altitude (Bergmann’s rule and the ConverseBergmann's rule[22] ~~(Fairbairn 1997)~~). Bergmann’s rule postulates that body size of mammals and insects increases with greater latitude[21-23] ~~(Tu et al. 2011, Fairbairn 1997, Deng and Xue 1997)~~, perhaps because of photoperiod influences ~~due to photoperiod~~[19] ~~(Amy and Nicholas 1999)~~. We speculate that the observed difference in body size between the two *A. mali* clades may be due to their occurrence in locations of different latitude~~(Liaoning clade: 122°31'E, 39.20°N, Shandong clade: 116°41'E, 39°91'N)~~.

Mitochondrial genome sequences are an important marker in molecular systematics that are often used in phylogenetic studies to resolve unclear evolutionary relationships among closely related insects[24-26] ~~(Castro and Dowton 2005, Cha et al. 2007, Sun et al. 2010)~~.

The AT and GC skew values reflect deviation from a population’s base composition,

which has important inference value for studying the mechanism of mitochondrial genome replication and transcription[27] ~~(Wei et al. 2010)~~. Skew values (AT: Shandong clade 0.1291, Liaoning clade 0.1305; GC:Shandong clade -0.1619, Liaoning clade -0.1328) of the *A.mali* clades were normal and in the general range for wasps[18]. The A+T content of other Hymenoptera is from about 76% to 86%[18] ~~(Wu et al. 2014)~~, and the values of ~~which was not significantly different from that of~~ these two clades of *A.mali* (Shandong clade: 84.44%, Liaoning clade: 83.99%) were within the range. ~~,~~P~~p~~resent technology cannot determine the complete sequence of the mitochondrial genome of some insects, including *A.mali*[28]~~(Du et al.2020)~~. The genetic measures we obtained suggest that the two clades of *A. mali* belonged to the same species.

Crossing experiments are commonly used to resolve species relationships in the parasitic Hymenoptera because laboratory cultures of these wasps are often maintained during biological control projects[10] ~~(Maxwell et al. 2008)~~. According to Pinto[16],relative reproductive compatibility index values that are below 0.75 suggest partial reproductive isolation among populations[19,20] ~~(Amy et al. 1999, Sun et al. 2007)~~, ~~Since~~ since the ~~re~~productive compatibility index values we obtained for the two *A. mali* clades (L♂×S♀: 1.188, S♂×L♀: 0.964) were far above this 0.75 value, we conclude that these crosses strongly suggest there is no mating incompatibility between these populations, and that there likely is significant gene exchange between the two clades[29,30] ~~(Herrero et al. 2018, Fogliata et al. 2016)~~. Indeed, previous studies have documented such gene flow between these populations, which is what

would be expected, given the small geographical distance between the two clades' distributions~~(Zhou et al. 2014)~~[12]. Generally speaking, the formation of species mainly includes the allopatric speciation and the sympatric speciation. Allopatric speciation is also called geographical speciation, which is considered by modern evolutionists to be the main way of population speciation into reproductive isolation species[31-33] ~~(Ayala 1976, Feder and Filchak 1999, Morgan et al. 2000)~~. ~~The process is that b~~Before ~~the beginning of~~ speciation, the environment is relatively uniform, the populations exist with a single or a series of similar form. Then, there are environmental differences and subgroups that adapt to each community. However, there was no reproductive isolation.

In this paper, we examined samples from two populations of *A. mali*, the Qingdao population of the Shandong clade and the Qinhuangdao population of the Liaoning clade. We found no reproductive isolation between these two populations. However, populations from more widely separated western or southern locations (e.g., in Xinjiang or Yunnan Provinces) of these clades may be more reproductively isolated and this possibility needs further clarification.

Acknowledgements

The authors are highly thankful to Roy Van Driesche for English and Scientific editing as well for suggestions and advice on this research. This work was supported

by the National Natural Science Foundation（31371994~~31772232~~) and the Taishan Mountain Scholar Constructive Engineering Foundation of Shandong, China.

References

1 Jaume L, Simó A, Ferran G, M. José S , Georgina A. Woolly apple aphid *Eriosoma lanigerum* Hausmann ecology and its relationship with climatic variables and natural enemies in Mediterranean areas. Bull Entomol Res 2015;105(1):60–69.

2 Zhang Q, Luo WC. Occurrence characteristics of *Eriosoma lanigerum* and its control methods. Entomol Knowledge. 2002;39(5):340–342.

3 Wang XY, Jiang CT, Xu GQ. Potential distribution ofan invasive pest *Eriosoma lanigerum* in China. Chinese Journal of Applied Entomology 2011; 48(2): 379–391.

4 Chen WM, Yu JN, Xu Y, Liu XL, Ru KY. The first occurrence of woolly apple aphid in Xinjiang. Xinjiang Agric Sci2006;4:309.

5 Zhou HX, Yu Y, Tan XM, Chen AD, Feng JG. Biological control of insect pests in apple orchards in China. BiolContr. 2014;68:47–56.

6 Yang QR, Gao ZM, Zheng Y, Wang T. Investigation on the natural enemies of woolly apple aphid. Plant Quarantine. 2005;19(1): 52-52.

7 Kuang RP, Shan F, Tang YZ, Zhong N. Evaluation of natural enemies of woolly apple aphid, *Aphelinus mali* (Hald). Natural Enemies of Insects.1985;11(2): 51-56.

8 Ma M, Guo JY, Tan XM, Sun LN, Zhou HX, Wan FH. *Aphelinus mali*—parasitic

Formatted: Font: Not Italic

Formatted: Font: Not Bold

Formatted: Font: Not Italic

Formatted: Font: Not Bold

Formatted: Font: Italic

Formatted: Font: 12 pt, Not Italic

Formatted: Font: 12 pt, Not Bold

Formatted: Font: 12 pt, Not Italic

Formatted: Font: 12 pt, Not Bold

Formatted: Font: 12 pt, Not Italic

Formatted: Font: 12 pt, Not Bold

Formatted: Font: 12 pt, Not Italic

Formatted: Font: 12 pt, Not Bold

wasps with potential to control *Eriosoma lanigerum*. Entomol J East China. 2008;17(1):72-73.

9 Long CD, Wang YP, Tang PZ. Studies on the biological characteristics and utilization of *Aphelinus mal*i Haldeman. Acta Entomol Sinica. 1960;10(1):1-39.

10 Maxwell KB, Susan WK, Robert AW, William AO, David DW, Millicent AC. Cross mating studies among five fruit fly parasitoid populations: potential biological control implications for tephritid pests. BioControl. 2008;53:709–724.

11 Zhang RM, Zhou HX, Guo D, Tao YL, Wan FH. Two putative bridgehead populations of *Aphelinus mali* (Hymenoptera: Aphelinidae) introduced in China. Fla.Entomol. 2014; 97(2):401-405.

12 Zhou HX, ZhangRM, GuoD, TaoYL, WanFH, Wu Q,et al. Analysis of genetic diversity and structure of two clades of *Aphelinus mali* (Hymenoptera: Aphelinidae) in China. Fla. Entomol. 2014;97(2), 699–706.

13 Su M, Tan XM, Yang QM, Zhou HX, Wan FH. Relative efficacy of two clades of *Aphelinus mali* (Hymenoptera: Aphelinidae) for control of woolly apple aphid (Hemiptera: Aphididae) in China. J. Econ.Entomol.2017;110:35–40.

14 Su M, Tan XM, Yang QM, Zhao CM, Wan FH,Zhou HX. Laboratory comparison of two *Aphelinus mali* clades for control of woolly apple aphid from Hebei Province. China. Bull.Entomol.Res.2018;108(3):400-405.

15 Su M, Zheng XM, Tan XM, Chu D, Zhou HX, Wan FH. Antennae characteristics of *Aphelinus mali* (Haldeman) male and female adults. Shandong Agri Sci.2016;48(7):106-109.

Formatted: Font: Not Italic
Formatted: Font: Not Bold
Formatted: Font: Not Italic
Formatted: Font: Not Italic
Formatted: Font: Not Bold
Formatted: Font: Not Italic
Formatted: Font: Not Bold
Formatted: Font: Not Italic
Formatted: Font: Not Italic
Formatted: Font: Not Italic
Formatted: Font: Not Bold
Formatted: Font: Not Italic
Formatted: Font: Not Bold
Formatted: Font: Not Italic
Formatted: Font: Not Bold
Formatted: Font: Not Italic
Formatted: Font: Not Bold

16 Pinto JD, Stouthamer R, Planter GR, Oatman ER. Variation in reproductive compatibility in *Trichogramma* and its taxonomic significance (Hymenoptera: Trichgrammatidae). Ann.Entomol.Soc. Am.1991; 84(1):37-46.

17 Shi ZH, Liu SS, Gebremeskel FB. Comparative study on reproductive compatibility of two geographic populations of *Diadromus collaris* (Gravenhors) (Hym.:Ichneumonidae),a pupal parasitoid of *Plutellaxylostella* (L.) (Lep.:Plutellidae). J Zhejiang Univ.2002;28(2):136-140.

18 Wu QL, Li Q, Gu Y, Shi BC. The complete mitochondrial genome of *Taeniogonalos taihorina* (Bischoff) (Hymenoptera: Trigonalyidae) reveals a novel gene rearrangement pattern in the Hymenoptera. Gene.2014; 543:76–84.

19 Arnett AE, Nicholas JG. Bergmann's rule in the ant lion *Myrmeleon immaculatus* DeGeer (Neuroptera: Myrmeleontidae): geographic variation in body size and heterozygosity. Journal of Biogeography.1999;26,275-283.

20 Sun L, He HM, Xue FS. Geographic Variation of Diapause in Insects. Acta Agriculturae Universitatis Jiangxiensis.2007;29(6):921-927.

21 Tu XY, Xia QW, Chen C, Chen YS, Kuang XJ, Xue FS. Geographic variation in body weight and size of the Asian corn borer, *Ostrinia furnacalis* Guenée (Lepidoptera: Pyralidae). Acta EntomologicaSinica.2011;54(2):143-148.

22 Fairbairn DJ. Allometry for sexual size dimorphism: pattern and process in the coevolution of body size in males and females. Annu Rev Ecol Syst.1997;28:659-687.

23 Deng T, Xue XX. An existing countermeasure of mammals in glacial periods—a

Formatted: Font: Not Italic

Formatted: Font: Not Italic

Formatted: Font: Not Italic

Formatted: Font: Not Bold

Formatted: Font: Not Italic

Formatted: Font: Not Italic

Formatted: Font: Not Italic

Formatted: Font: Not Italic

Formatted: Font: Not Bold

Formatted: Font: Not Italic

Formatted: Font: Not Bold

Formatted: Font: Not Italic

Formatted: Font: Not Bold

Formatted: Font: Not Italic

Formatted: Font: Not Bold

new explanation to the Bergmann's Law. Acta Theriologica Sinica. 1997;17(4):259-265.

24 Castro LR, Dowton M. The position of the Hymenoptera with in the Holometabola as inferred from the mitochondrial genome of *Perga condei* (Hymenoptera: Symphyta: Pergidae). Mol Phyl Evol. 2005;34(3):469-479.

25 Cha SY, Yoon HJ, Lee EM, Yoon MH, Hwang JS, Jin BR, et al. The complete nucleotide sequence and gene organization of the mitochondrial genome of the bumblebee, *Bombus ignitus* (Hymenoptera: Apidae). Gene.2007;392(1 -2):206-220.

26 Sun Z, Zhang J, Wang R, Xu YJ, Zhang DQ. Progress of insect mitochondrial genome. J Inspection and Quarantine.2010;20(03):69-73.

27 Wei SJ, Shi M, Chen XX, Sharkey MJ, Van AC, Ye CY,et al. New views on strand asymmetry in insect mitochondrial genomes. PloS ONE. 2010; 5:1767-1780.

28 Du LJ, Wei SJ, Tan XM, Chu XX, Yang QM, Wang JQ, et al. Mitochondrial genome of *Aphelinus mali* (Haldeman) (Hymenoptera: Aphelinidae) with a higher AT percent and the highest AT-skew in Hymenoptera insects. Entomol. News.2020;129(3):267-285.

29 Herrero MI, Fogliata SV, Vera A, Casmuz A, Sosa GD, Castagnaro AP, et al. Biological characterization and mating compatibility of *Helicoverpa gelotopoeon* (D.) (Lepidoptera: Noctuidae) populations from different regions in Argentina. Bull Entomol Res.2018;108(1):108-115.

30 Fogliata SV,Vera A, Gastaminza G, Cuenya MI, Zucchi MI, Willink E, et al. Reproductive isolation between two populations of *Diatraea saccharalis* (F.) (Lepidoptera: Crambidae) from different host plant species and regions in Argentina. Bull Entomol Res.2016;106(5):591-597.

31 Ayala FJ. Genetic differentiation during the speciation process. Evolutionay Biology.1975;8:1–75.

32 Feder JL, Filchak KE. It's about time: the evidence for host plant-mediated selection in the apple maggot fly, *Rhagoletis pomonella*, and its implications for fitness trade-offs in phytophagous insects. Entomol Exp Appl. 1999;91(1): 211–225.

33 Morgan DJW, Reitz SR, Atkinson PW, Trumble JT. The resolution of Californian population of *Liriomyza huidobrensis* and *Liromyza trifolii* (Diptera: Agromyzidae) using PCR. Heredity.2000;85(1): 53–61.

Formatted: Font: Not Italic
Formatted: Font: Not Bold
Formatted: Font: Not Italic
Formatted: Font: Not Bold
Formatted: Font: Not Italic
Formatted: Font: Not Bold
Formatted: Font: Not Italic
Formatted: Font: Not Bold

Table 1. Primers used for amplification of the mitochondrial genome

Formatted Table

**Fragment No.**	**Gene or region**	**Primer name**	**Sequence (5’-3’)**	**Length(bp)**
F1	*tRNA-Cys-COX3*	RGF1	CCAAWTAAGAAATATAAATATTC	871
		RGR1	CTGGTTTTCATGGTCTTCATG	
F2	*COX3*	RGF2	GTCAATATCAAGCTGCAGC	191
		RGR2	AGAGTATTCTGAAGCTTC	
F3	*COX3-ATP6*	RGF3	GTTCCTATTAAAACATGAAGAC[illegible]	926
		RGR3	CCTTTAGGGACACCAGGAG	
F4	*ATP6*	RGF4	GACCAGCAATCATATTTGCAG	486
		RGR4	CTGTTTTTGATCCTTCTAC	
F5	*ATP6-COX1*	RGF5	ATAACGAGAAGGAATAAATC	1876
		RGR5	GAATGGATGTAGATACTCG	
F6	*COX1*	RGF6	CCATTTATTGAAGCAAGTCATC	616
		RGR6	CTGGGACAGGTTGAACTG	
F7	*COX1-NAD5*	RGF7	TTGAACCTATAATTGAAG	1346
		RGR7	GTCAAGATATTCGTTTTATAGG	
F8	*NAD5*	RGF8	AAGGTATTCCACATAATG	710
		RGR8	TGGGTGGGATGGTTTAGG	
F9	*NAD5-NAD4*	RGF9	TATTACATCTCCAATTCG	1073
		RGR9	GTTTGTTTACGTCAGTATG	
F10	*NAD4*	RGF10	GCTGAAGAACATAAACCATG	331
		RGR10	GACTTCCTAAAGCTCATG	
F11	*NAD4-CYTB*	RGF11	CGAACAACACCATAACCC	2239
		RGR11	GAATAAAATCGATTTAGTGTAGC	
F12	*CYTB*	RGF12	TGAGGAGCTACTGTTATTAC	396
		RGR12	TCATTCAGGTTGAATATG	
F13	*CYTB-NAD1*	RGF13	CCATATATTTTAAGAGACCC	1475
		RGR13	GATTTATAATAATTAGGTTTCCA[illegible]	
F14	*NAD1*	RGF14	CCTTTCATATATTCTGTG	397

		RGR14	GAAATAATTTACCATTTTATTCT[illegible]	
F15	*NAD1-RRNL*	RGF15	GAGCAATACTCCGAAGAC	697
		RGR15	GTTTGCGACCTCGATGTTG	
F16	*RRNL*	RGF16	CTCACGCCGATCTAAACTC	1051
		RGR16	CCTTTTGTATCAGGGTTA	
F17	*RRNL-RRNS*	RGF17	TTAATCATTGAGCAGATC	871
		RGR17	GTACAAATTGCCCGTCGC	
F18	*RRNS*	RGF18	ACTATATTCCTAACTTCT	461
		RGR18	AACTAGGATTAGATACC	
F19	*RRNS-tRNA-Asn*	RGF19	CAATTACAAAACAAGTTCCTC	904
		RGR19	CTTAATTGGAAAAATAATTTC	

Table12 Morphological measurements of two clades of *Aphelinus mali* in China. Data are mean± SD(μ m), anddifferent letters in the same row indicate significant differences.

Character length		Females (Mean ± SD)		Males (Mean ± SD)	
		Shandong	Liaoning	Shandong	Liaoning
Whole body		810.93 ± 16.03b	870.29 ± 12.18a	679.34 ± 12.34d	758.38 ± 15.95c
Abdomen		384.12 ± 9.75b	424.43 ± 7.87a	292.96 ± 7.53d	339.94 ± 8.04c
Forewing		682.40 ± 11.09c	770.86 ± 9.39a	609.84 ± 8.42d	735.96 ± 8.35b
Hind wing		303.46 ± 4.99c	336.84 ± 4.14a	277.70 ± 4.02d	321.52 ± 3.51b
Anterior leg	Femur	176.70 ± 3.37c	195.25 ± 2.52a	167.46 ± 2.14d	188.30 ± 2.24b
	Tibia	147.32 ± 2.67b	163.43 ± 2.29a	138.67 ± 2.20c	159.98 ± 2.36a
	Tarsus	158.73 ± 2.87b	168.58 ± 2.87a	150.56 ± 2.18c	166.04 ± 2.48a
	Pre-tarsus	19.36 ± 0.41b	24.14 ± 1.36a	19.54 ± 0.46b	23.31 ± 0.38a
	Spur	26.23 ± 0.96bc	27.56 ± 0.56b	25.72 ± 0.71c	29.17 ± 0.76a
	Total leg	528.32 ± 8.42b	551.39 ± 6.96a	476.22 ± 6.33c	543.33 ± 9.01a
Middle leg	Femora	214.75 ± 3.14c	238.31 ± 3.11a	200.52 ± 3.22d	228.08 ± 2.85b
	Tibia	249.91 ± 4.53c	276.08 ± 3.51a	229.60 ± 3.50d	265.51 ± 3.05b
	Tarsus	202.98 ± 3.74b	215.30 ± 3.24a	191.74 ± 2.87c	210.43 ± 3.03a
	Pre-tarsus	19.36 ± 0.41b	23.26 ± 0.29a	19.87 ± 0.42b	22.82 ± 0.29a
	Spur	66.91 ± 1.67b	74.05 ± 1.15a	66.27 ± 1.40b	72.19 ± 2.87a
	Total leg	753.51 ±12.23a	752.95 ± 9.44a	641.73 ± 9.09c	728.55 ± 10.62b
Hind leg	Femora	253.72 ± 4.56a	256.15 ± 3.75a	223.63 ± 3.44c	239.85±2.87b
	Tibia	259.67±4.06c	288.79±4.02a	231.29 ± 3.68d	274.67 ± 3.75b
	Tarsus	226.86 ± 4.23b	242.70 ± 3.72a	211.99 ± 3.41c	233.09 ± 3.30b
	Pretarsus	19.79 ± 0.42b	23.30 ± 0.34a	19.64 ± 0.44b	23.24 ± 0.32a
	Spur	32.20 ± 1.16c	36.54 ± 0.73a	28.60 ± 0.92d	34.16 ± 0.68b
	Total leg	792.33 ± 12.32a	810.94 ± 11.57a	686.55 ± 9.61c	768.52 ± 12.10b
Antenna	Funicle1	16.52 ± 0.44b	19.03 ± 0.31a	16.27 ± 0.43b	18.96 ± 0.32a
	Funicle2	18.04 ± 0.30b	19.33 ± 0.30a	16.44 ± 0.29c	18.94 ± 0.30a
	Funicle3	33.12 ± 0.44c	36.57 ± 0.54b	46.83 ± 0.76a	45.18 ± 1.52a
	Club	91.13 ± 1.37d	106.76 ± 1.03b	100.10 ± 2.19c	113.90 ± 1.72a
	Total antenna	158.81 ± 2.18c	181.68 ± 1.68b	179.62 ± 2.92b	212.50 ± 2.55a

Data are mean ± SD (μ m), and different letters in the same row indicate significant differences.

Table ~~2~~3 Comparisons of the mitochondrial genome between two clades of *Aphelinus mali* in China

G~~ENE~~ene	Clade	Strand	Length(bp）	Anti/start codon	Stop codon	Intergenic nucleotides
COX3	Shandong	-	732	ATG	TAA	39
	Liaoning	-	732	ATG	TAA	2
ATP6	Shandong	-	651	ATT	TAA	3
	Liaoning	-	651	ATT	TAG	-10
ATP8	Shandong	-	156	ATT	TAA	-10
	Liaoning	-	162	ATT	TAA	24
TRND	Shandong	-	68	GTC		24
	Liaoning	-	71	GTC		10
TRNK	Shandong	+	70	TTT		9
	Liaoning	+	74	TTT		2
COX2	Shandong	-	660	ATT	TAA	2
	Liaoning	-	660	ATT	TAA	0
TRNL2	Shandong	-	68	TAA		0
	Liaoning	-	67	TAA		14
COX1	Shandong	-	1512	ATT	TAA	-18
	Liaoning	-	1509	ATT	TAA	-18
TRNE	Shandong	+	67	TTC		4
	Liaoning	+	67	TTC		-1
TRNF	Shandong	-	70	GAA		-1
	Liaoning	-	67	GAA		0
NAD5	Shandong	-	1479	ATT	TAA	11
	Liaoning	-	1563	ATT	T	12
TRNH	Shandong	-	68	GTG		0
	Liaoning	-	68	GTG		0
NAD4	Shandong	-	1335	ATG	T	-7
	Liaoning	-	1308	ATG	T	-7
NAD4L	Shandong	-	273	ATA	TAA	-7
	Liaoning	-	267	ATA	TAA	-7
TRNT	Shandong	+	66	TGT		0
	Liaoning	+	66	TGT		
TRNP	Shandong	-	65	TGG		2
	Liaoning	-	64	TGG		
NAD6	Shandong	+	519	ATA	T	15
	Liaoning	+	513	ATA	T	1

Gene	Clade	Strand	Length(bp）	Anti/start codon	Stop codon	Intergenic nucleotides
COB	Shandong	+	1083	ATA	TAA	1
	Liaoning	+	1083	ATA	TAA	27
TRNS2	Shandong	+	65	TGA		27
	Liaoning	+	65	TGA		73
TRNS2	Shandong	+	65	TGA		77
	Liaoning	+	70	TGA		77
TRNS2	Shandong	+	65	GGA		78
	Liaoning	+	65	GGA		77
TRNS2	Shandong	+	65	TGA		149
	Liaoning	+	65	TGA		149
NAD1	Shandong	-	891	ATA	TAA	0
	Liaoning	-	891	ATA	TAA	0
TRNL1	Shandong	-	66	TAG		0
	Liaoning	-	66	TAG		0
RRNL	Shandong	-	1359			0
	Liaoning	-	1360			0
TRNA	Shandong	-	70	TGC		0
	Liaoning	-	67	TGC		0
RRNS	Shandong	-	769			0
	Liaoning	-	771			0
TRNV	Shandong	-	56	TAC		323
	Liaoning	-	56	TAC		20
NAD2(P)	Shandong	-	240	ATT	ATT	40
	Liaoning	-	213	ATT	TGA	
TRNN	Shandong	+	66	GTT		0
	Liaoning	+	66	GTT		
TRNC	Shandong	-	64	GCA		
	Liaoning	-	64	GCA		44
TRNR	Shandong	-	59	TCG		
	Liaoning	-	59	TCG		2
NAD3	Shandong	-	312	ATA	TAA	
	Liaoning	-	312	ATA	TAA	-3
TRNG	Shandong	-	65	TCC		
	Liaoning	-	65	TCC		41
//	Shandong		998			
	Liaoning		972			

~~Table~~ Table~~3~~ 4 The base content, AT skewness and GC skewness of the two *Aphelinus mali* clades compared to other Hymenoptera

Species	T%	C%	A%	G%	A+T%	ATskew	GCskew
Aphelinus mali SC	36.69	9.40	47.30	6.61	84.44	0.1291	-0.1619
Aphelinus mali LC	36.69	9.40	47.30	6.61	83.99	0.1305	-0.1328
Taeniogonalos taihorina	43.51	6.77	41.09	8.63	84.60	-0.0286	0.1208
Spathius agrili	45.12	6.55	38.88	9.45	84.00	-0.0743	0.1813
Cotesia vestalis	47.46	5.78	39.70	7.06	87.16	-0.0890	0.0997
Enicospilus sp.	43.44	8.73	41.45	6.08	85.19	-0.0198	-0.1789
Abispa ephippium	39.50	14.60	39.10	6.70	78.60	-0.0051	-0.3709
Bombus hypocrita	42.56	9.68	42.77	4.98	85.33	0.0025	-0.0275
Perga condei	33.80	14.60	42.80	8.80	76.60	0.1175	-0.2479

SC: Shandong clade, LC: Liaoning clade

Formatted Table

Table ~~4~~5 The base composition of mitochondrial genomic coding gene and rRNA gene of two clades of *Aphelinus mali* in China

Gene~~ENE~~	Clades	T%	C%	A%	G%	A+T%	AT skew	GC skew
COX3	Shandong	30.13	12.05	49.23	8.59	79.36	0.2454	-0.1676
	Liaoning	29.87	12.44	49.10	8.59	78.97	0.2435	-0.1831
ATP6	Shandong	30.83	8.85	50.88	9.44	81.71	0.2454	-0.0323
	Liaoning	31.56	9.29	50.15	9.00	81.71	0.2282	-0.0159
ATP8	Shandong	42.42	4.85	49.09	3.64	91.52	0.0729	-0.1427
	Liaoning	41.82	4.85	49.09	4.24	90.91	0.0800	-0.0671
COX2	Shandong	34.51	11.01	46.26	8.22	80.76	0.1455	-0.1450
	Liaoning	34.80	11.01	45.81	8.37	80.62	0.1366	-0.1362
COX1	Shandong	31.66	12.81	45.75	9.78	77.41	0.1820	-0.1341
	Liaoning	30.82	13.32	46.27	9.59	77.09	0.2004	-0.1628
NAD5	Shandong	33.21	.72	52.21	5.85	85.42	0.2224	-0.1968
	Liaoning	33.13	8.49	52.63	5.74	85.77	0.2274	-0.1933
NAD4	Shandong	34.28	8.79	50.67	6.26	84.95	0.1929	-0.1681
	Liaoning	32.11	11.60	47.01	9.28	79.12	0.1883	-0.1111
NAD4L	Shandong	34.01	8.42	54.55	3.03	88.55	0.2320	-0.4707
	Liaoning	35.35	7.74	54.88	2.02	90.23	0.2164	-0.5860
NAD6	Shandong	45.82	6.00	44.91	3.27	90.73	-0.0100	-0.2945
	Liaoning	44.91	6.79	45.06	3.24	89.97	0.0017	-0.3539
COB	Shandong	43.73	10.93	35.29	10.04	79.02	-0.1068	-0.0424
	Liaoning	42.81	11.58	35.53	10.09	78.33	-0.0929	-0.0688
NAD1	Shandong	33.76	11.75	47.44	7.05	81.20	0.1685	-0.2500
	Liaoning	33.44	12.39	47.22	6.94	80.66	0.1708	-0.2818
NAD3	Shandong	33.33	9.32	52.26	5.08	85.59	0.2212	-0.2942
	Liaoning	33.33	9.60	51.41	5.65	84.75	0.1661	-0.2590
RRNL	Shandong	41.20	7.46	46.76	4.57	87.97	0.0632	-0.2352
	Liaoning	41.08	7.55	46.95	4.42	88.03	0.0667	-0.2615
RRNS	Shandong	42.39	7.67	46.42	3.51	88.82	0.0454	-0.3721
	Liaoning	42.15	7.65	46.82	3.37	88.98	0.0525	-0.3884
NAD2	Shandong	35.00	5.00	55.00	5.00	90.00	0.2222	0
	Liaoning	35.92	4.92	54.93	4.23	90.85	0.2092	-0.0754

Table ~~5~~6 Results of crosses between the two *Aphelinus mali* clades, in terms of the number of pupae, progeny produced, emergence rate, female ratio and relative compatibility of hybridization. ~~Data in the same row are means ± SD, different letters indicate significant differences (*P*<0.05). L: Liaoningclade.S: Shandong clade~~

	# pupae	# adult progeny	% emergence	Sex ratio (%F)	relative compatibility
L♂×S♀（n=18）	5.28 ± 2.93a	4.67 ± 3.03ab	85.09± 16.75a	0.57 ± 0.28a	1.188
S♂×L♀（n=15）	4.86 ± 4.28a	3.78 ± 3.62a	77.94 ± 28.87a	0.53 ± 0.32a	0.964
S♂×S♀（n=15）	7.00 ± 4.95a	6.37 ± 1.69ab	91.00 ± 13.98a	0.48 ± 0.33a	——
L♂×L♀（n=18）	8.67 ± 2.89a	7.29 ± 2.39b	84.08± 30.29a	0.55 ± 0.34a	——
	df=3 F=1.147	df=3 F=2.058	df=3 F=1.076	df=3 F=0.133	
	P=0.343	P=0.123	P=0.372	P=0.940	

Data in the same column are means ± SD, different letters indicate significant differences (*P*<0.05). L: Liaoning clade. S: Shandong clade

Formatted Table

Formatted: Line spacing: Double

Data in the same row are means ± SD, different letters indicate significant differences (*P*<0.05). L: Liaoningclade.S: Shandong clade

Table 67 The number of pupae, progeny produced, emergence rate, sex ratio (as %F)and relative compatibility of backcross. Data in the same row are mean ± SD, different letters indicate significant differences (*P*<0.05). L: Liaoningclade;S: Shandong clade.

	S# pupae	# progeny	% emergence	Sexratio (% F)	relative compatibility
(S♀×L♂) ♂backcrossS♀ (n=5)	3.80 ± 2.95a	3.40 ± 3.88b	77.50 ± 43.66a	0.47 ± 0.35a	0.980
(S♀×L♂) ♀backcrossL♂ (n=3)	4.33 ± 1.53a	4.00 ± 1.00ab	94.40 ± 9.62a	0.59 ± 0.08a	1.230
(S♂×L♀) ♂backcrossL♀ (n=3)	3.33 ± 1.53a	3.00 ± 2.00b	83.30 ± 28.87a	0.76 ± 0.21a	1.382
(S♂×L♀) ♀backcrossS♂ (n=3)	6.67 ± 2.08a	6.33 ± 2.52ab	93.30 ± 11.55a	0.61 ± 0.10a	1.110
S♂×S♀ (n=15)	7.00 ± 4.95a	6.37 ± 1.69ab	91.00 ± 13.98a	0.48 ± 0.33a	——
L♂×L♀ (n=18)	8.67 ± 2.89a	7.29 ± 2.39a	84.08± 30.29a	0.55 ± 0.34a	——
	df=5 F=1.488	df=5 F=1.829	df=5 F=0.386	df=5 F=0.615	
	P=0.249	P=0.164	P=0.851	P=0.690	

Data in the same column roware mean ± SD, different letters indicate significant differences (*P*<0.05). L: Liaoning clade; S: Shandong clade.

Formatted: Line spacing: single, Widow/Orphan control

Formatted Table

Formatted: Indent: Left: 0", Right: 0", Line spacing: Double

Formatted: Indent: Left: 0", Right: 0"

1. In the Methods section, we add the additional location information of the collection site, including geographic coordinates.

2. No permits were required in our work, because we collect the samples in the orchards which with no management.

3. We have added the amended Role of Funder statement in our cover letter. And we changed a funder (31772232) to a new one (31371994).

The funder "National Natural Science Foundation (31371994)" play a role in study design, data collection and preparation of the manuscript, the funder " Taishan Mountain Scholar Constructive Engineering Foundation of Shandong, China " play a role in data collection.

Reviewer #1:

1. Why the population collected in Hebei can represent the Liaoning clade, but not the Shandong clade.

Because in our previous research has found that the *A.mali* in Qinhuangdao (Hebei province) belong to Liaoning clade. [Zhang RM, Zhou HX, Guo D, Tao YL, Wan FH. Two putative bridgehead populations of *Aphelinus mali* (Hymenoptera: Aphelinidae) introduced in China. Fla.Entomol. 2014; 97(2):401-405]

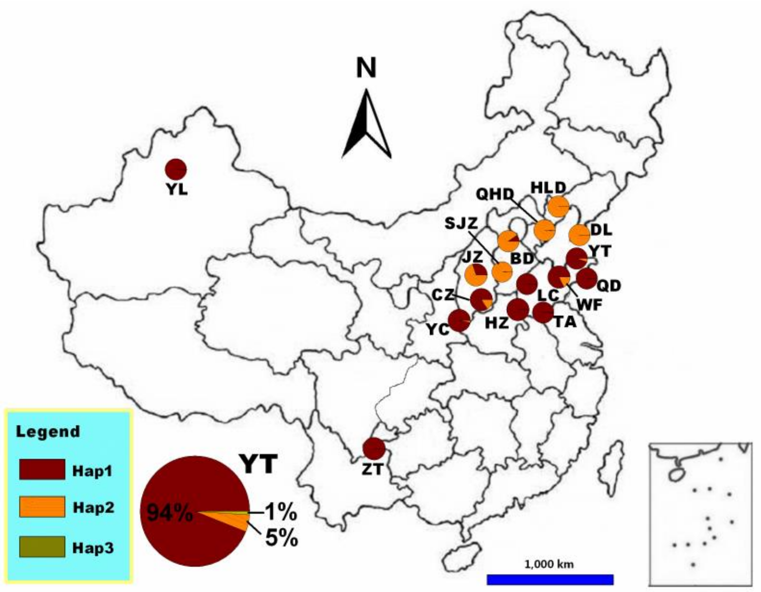

2. Geographic information about Liaoning and Shandong should first be provided in the introduction.

The geographic information now has been provided in the introduction.

3. The formatting and language issues have been improved.

4.The minor suggestions have been revised.

Reviewer #2:

1. About the Order and Family of the wasps.

We are so sorry about that, we were careless in our writing, we have revised in the manuscript.

2. About the authors' name.

The correct order we have revised in the manuscript.

3.We have provided a map in the introduction to show the geographic information about Liaoning and Shandong.

4. How we were maintained the colony.

We choose an apple orchard with less management and protected the apple trees that had colonies of *E. lanigerum* with screen cages (1m×1m×2m). Thereafter, we pick out the parasitized black aphids daily until it doesn't appear, make sure there were no *A. mali* in the *E. lanigerum* colony. The cages can keep the predators out.

5. Due to our carelessness, we forgot to change the instructions when we changed the table format.

6. We have modified the part of References followed the journal's formatting guidelines.

7. The amendments in the PDF have been revised.

line 31: We mean there were no significant differences between the "leg" section.

Reviewer #3:

1. Abstract

We have revised the part of the abstract.

2. Introduction

We have revised the part of the introduction. The reference [Su et al.2008] focused on the comparison of two clades of the *A. mali*, in the text, we use the *E. lanigerum* from Hebei Province as the host.

3. M&M

About the primer: The sequences of the primers were not provided in the manuscript, because the mitochondrial genome is so large, we designed the primer while we cloned it, and then amplified the full length, so it's a lot of primers.

About the screen cage: We used a very big screen (a net) to cover the apple tree to protect the host.

About the sample: In our previous research has found that the *A.mali* in Qinhuangdao (Hebei province) belong to Liaoning clade. [Zhang RM, Zhou HX, Guo D, Tao YL, Wan FH. Two putative bridgehead populations of *Aphelinus mali* (Hymenoptera: Aphelinidae) introduced in China. Fla.Entomol. 2014; 97(2):401-405]

About the amounts of insect in each repeat: Two insects (one male and one female) in each repeat.

About the ANOVA: Significant differences were determined using

one-way analysis of variance(ANOVA) corrected by SPSS 19.0. The independent samples t-test was used to analyze the data on parthenogenesis between two clades, using SPSS 19.0.

Discussion:

1. The mitochondrial genomes structure of these two clades were incompleted.

2. Although the latitude of these two locations was very close, they were belong to different clade refer to our previous research.[Zhang RM, Zhou HX, Guo D, Tao YL, Wan FH. Two putative bridgehead populations of *Aphelinus mali* (Hymenoptera: Aphelinidae) introduced in China. Fla.Entomol. 2014; 97(2):401-405]

3. In this research , we compared the morphological characteristics first and found some differences between two clades of *A.mali*, so we analyzed the mitochondrial genome sequence.

4. The range was refered to the research [Wu QL, Li Q, Gu Y, Shi BC. The complete mitochondrial genome of *Taeniogonalos taihorina* (Bischoff) (Hymenoptera: Trigonalyidae) reveals a novel gene rearrangement pattern in the Hymenoptera. Gene.2014; 543:76–84]

5. We think that there likely is gene change between two clades of *A.mali*, because there are many significant differences between two clades such as the morphology, relative efficacy and control ability, but there is no mating incompatibility.

6. For the cross-mating experiment, we collected *A.mali* as parasitized aphids on apple trees (as described below) from Qingdao (120°39E, 36°31N) to represent the Shandong clade and from Qinhuangdao (119°48E, 39°83N) from the Liaoning clade.

The minor suggestions have been revised.

Reviewer #4:

12. About the age of the parasitoids: We used the aphids mixed older and young as the host, and the parasitoids were adult age.

14. About the criteria followed to choose a sample: We choose 50 males and 50 females from each sample area randomly.

16. This part **Mitochondrial genome sequencing** has been rewrite. We collected the *A.mali* samples and then sent to the Wuhan Transduction Bio Co., LTD for sequencing.

29. We didn't recorded the longevity and other fitness aspects relative to parental population, so we plan to compared populations from more widely separated western or southern locations about many other characters between two clades.

The minor suggestions have been revised.

Reviewer #5:

1. Both of these two clades of *A.mali* have no other host other than *E. lanigerum*.

2. The locations of samples from two clades of *A.mali* in our paper were nearly, so we plan to clarify populations from more widely separated western or southern locations (e.g., in Xinjiang or Yunnan Provinces) of these clades.

3. We didn't compared the taxonomic characters such as length of ovipositor, coloration of scape pedicel and flagellum as whole. But we plan to compared populations from more widely separated western or southern locations (e.g., in Xinjiang or Yunnan Provinces) of these clades.

The minor suggestions have been revised.
