# Supplementary material for: Differences in morphology, mitochondrial genomes, and reproductive compatibility between two clades of parasitic wasps Aphelinus mali (Hymenoptera: Aphelindae) in China
Source: PLoS One. 2023 Feb 2;18(2):e0279663. doi: 10.1371/journal.pone.0279663 (PMC9894431; doi:10.1371/journal.pone.0279663)
Supplement: Attachment — Submitted filename: rebuttal letter.docx [file pone.0279663.s008.docx]

1. In the Methods section, we add the additional location information of the collection site, including geographic coordinates.

2. No permits were required in our work, because we collect the samples in the orchards which with no management.

3. We have added the amended Role of Funder statement in our cover letter. And we changed a funder (31772232) to a new one (31371994).

The funder "National Natural Science Foundation (31371994)" play a role in study design, data collection and preparation of the manuscript, the funder " Taishan Mountain Scholar Constructive Engineering Foundation of Shandong, China " play a role in data collection.

**Reviewer #1:**

1. Why the population collected in Hebei can represent the Liaoning clade, but not the Shandong clade.

Because in our previous research has found that the *A.mali* in Qinhuangdao (Hebei province) belong to Liaoning clade. [Zhang RM, Zhou HX, Guo D, Tao YL, Wan FH. Two putative bridgehead populations of *Aphelinus mali* (Hymenoptera: Aphelinidae) introduced in China. Fla.Entomol. 2014; 97(2):401-405]

2. Geographic information about Liaoning and Shandong should first be provided in the introduction.

The geographic information now has been provided in the introduction.

3. The formatting and language issues have been improved.

4.The minor suggestions have been revised.

**Reviewer #2:**

1. About the Order and Family of the wasps.

We are so sorry about that, we were careless in our writing, we have revised in the manuscript.

2. About the authors' name.

The correct order we have revised in the manuscript.

3.We have provided a map in the introduction to show the geographic information about Liaoning and Shandong.

4. How we were maintained the colony.

We choose an apple orchard with less management and protected the apple trees that had colonies of *E. lanigerum* with screen cages (1m×1m×2m). Thereafter, we pick out the parasitized black aphids daily until it doesn't appear, make sure there were no *A. mali* in the *E. lanigerum* colony. The cages can keep the predators out.

5. Due to our carelessness, we forgot to change the instructions when we changed the table format.

6. We have modified the part of References followed the journal's formatting guidelines.

7. The amendments in the PDF have been revised.

line 31: We mean there were no significant differences between the "leg" section.

**Reviewer #3:**

1. Abstract

We have revised the part of the abstract.

2. Introduction

We have revised the part of the introduction. The reference [ Su et al.2008] focused on the comparison of two clades of the *A. mali*, in the text, we use the *E. lanigerum* from Hebei Province as the host.

3. M&M

About the primer: The sequences of the primers were not provided in the manuscript, because the mitochondrial genome is so large, we designed the primer while we cloned it, and then amplified the full length, so it’s a lot of primers.

About the screen cage: We used a very big screen (a net) to cover the apple tree to protect the host.

About the sample: In our previous research has found that the *A.mali* in Qinhuangdao (Hebei province) belong to Liaoning clade. [Zhang RM, Zhou HX, Guo D, Tao YL, Wan FH. Two putative bridgehead populations of *Aphelinus mali* (Hymenoptera: Aphelinidae) introduced in China. Fla.Entomol. 2014; 97(2):401-405]

About the amounts of insect in each repeat: Two insects (one male and one female) in each repeat.

About the ANOVA: Significant differences were determined using one-way analysis of variance(ANOVA) corrected by SPSS 19.0. The independent samples t-test was used to analyze the data on parthenogenesis between two clades, using SPSS 19.0.

Discussion:

1. The mitochondrial genomes structure of these two clades were incompleted.

2. Although the latitude of these two locations was very close, they were belong to different clade refer to our previous research.[ Zhang RM, Zhou HX, Guo D, Tao YL, Wan FH. Two putative bridgehead populations of *Aphelinus mali* (Hymenoptera: Aphelinidae) introduced in China. Fla.Entomol. 2014; 97(2):401-405]

3. In this research , we compared the morphological characteristics first and found some differences between two clades of *A.mali*, so we analyzed the mitochondrial genome sequence.

4. The range was refered to the research [Wu QL, Li Q, Gu Y, Shi BC. The complete mitochondrial genome of *Taeniogonalos taihorina* (Bischoff) (Hymenoptera: Trigonalyidae) reveals a novel gene rearrangement pattern in the Hymenoptera. Gene.2014; 543:76–84]

5. We think that there likely is gene change between two clades of *A.mali*, because there are many significant differences between two clades such as the morphology, relative efficacy and control ability, but there is no mating incompatibility.

6. For the cross-mating experiment, we collected *A.mali* as parasitized aphids on apple trees (as described below) from Qingdao (120°39E, 36°31N) to represent the Shandong clade and from Qinhuangdao (119°48E, 39°83N) from the Liaoning clade.

The minor suggestions have been revised.

**Reviewer #4:**

12. About the age of the parasitoids: We used the aphids mixed older and young as the host, and the parasitoids were adult age.

14. About the criteria followed to choose a sample: We choose 50 males and 50 females from each sample area randomly.

16. This part **Mitochondrial genome sequencing** has been rewrite. We collected the *A.mali* samples and then sent to the Wuhan Transduction Bio Co., LTD for sequencing.

29. We didn't recorded the longevity and other fitness aspects relative to parental population, so we plan to compared populations from more widely separated western or southern locations about many other characters between two clades.

The minor suggestions have been revised.

**Reviewer #5:**

1. Both of these two clades of *A.mali* have no other host other than *E. lanigerum*.

2. The locations of samples from two clades of *A.mali* in our paper were nearly, so we plan to clarify populations from more widely separated western or southern locations (e.g., in Xinjiang or Yunnan Provinces) of these clades.

3. We didn't compared the taxonomic characters such as length of ovipositor, coloration of scape pedicel and flagellum as whole. But we plan to compared populations from more widely separated western or southern locations (e.g., in Xinjiang or Yunnan Provinces) of these clades.

The minor suggestions have been revised.
