# Supplementary material for: Differences in morphology, mitochondrial genomes, and reproductive compatibility between two clades of parasitic wasps Aphelinus mali (Hymenoptera: Aphelindae) in China
Source: PLoS One. 2023 Feb 2;18(2):e0279663. doi: 10.1371/journal.pone.0279663 (PMC9894431; doi:10.1371/journal.pone.0279663)
Supplement: Attachment — Submitted filename: 6-9 rebuttal letter.docx [file pone.0279663.s010.docx]

1. We made the revision on the affiliation because of the official change of our research center. We revised “Key Laboratory of Integrated Plant Disease and Pest Control” to “Shandong Engineering Research Center for Environment-Friendly Agricultural Pest Management”.

2. We have added the amended Role of Funder statement in our cover letter. And we changed a funder (31772232) to a new one (31371994).

The funder "National Natural Science Foundation (31371994)" play a role in study design, data collection and preparation of the manuscript, the funder " Taishan Mountain Scholar Constructive Engineering Foundation of Shandong, China " play a role in data collection.

**Reviewer #1:**

1. Why the population collected in Hebei can represent the Liaoning clade, but not the Shandong clade.

We added “(Fig. 1)” at the end of the Cross-mating experiment part, to clarify why the Qinhuangdao population belongs to the Liaoning clade.

2. The other suggestions have been revised.

**Reviewer #2:**

The suggestions have been revised

**Reviewer #3:**

1. Abstract

We have changed some key words.

2. Results

Sorry about that, the descriptions of the A+T content were wrong. We have revised "A+T content of both clades were within the range of other Hymenoptera". The AT skew of both clades have higher values.

3. About the sample:

As mentioned, *Aphelinus mali* was introduced into China twice: the first introduction was from Japan into Dalian and Lvshun, Liaoning Province (122°31'E, 39.20°N) in 1942, while the second was from the former Soviet Union in 1950 into Qingdao, Shandong Province (116°41'E, 39°91'N). In our previous research, the *A.mali* in China belong to two clades: Shandong clade and Liaoning clade, and the two clades were very possible concerned with the introduced.

4. The other suggestions have been revised.

**Reviewer #4:**

Methods:

1. Because of we measured too many indexes of the *A. mali*, so we should select a large number of samples to be sure the diversity of samples.

2. We collected the insect samples and then sent to Wuhan Transduction Bio Co., LTD for extraction and detection of Genomic DNA, Primer design, LA-PCR amplification and sequencing, and then we analyzed the date.

Results：

1. The references of Table 3 (now Table 4) is [18].

2. "The number of hosts attacked….." is " the number of the pupae in the Table 6.

3. The map for the sampling sites was cited the reference [11]. The images of parasitoids were the came from our experiment.

4. The other suggestions have been revised.
