# Supplementary material for: Differences in morphology, mitochondrial genomes, and reproductive compatibility between two clades of parasitic wasps Aphelinus mali (Hymenoptera: Aphelindae) in China
Source: PLoS One. 2023 Feb 2;18(2):e0279663. doi: 10.1371/journal.pone.0279663 (PMC9894431; doi:10.1371/journal.pone.0279663)
Supplement: Attachment — Submitted filename: comments.docx [file pone.0279663.s011.docx]

1.	*Aphelinus mali* was introduced from Japan and the former Soviet Union into China. The different population sources may divided the parasitoid into two clades. For their morphology, the body size of Liaoning is larger than Shandong as shown in Table 2. Are there other different characters between two clades? Such as body color, wing shade, veins, ect., because at least we know that some species of the genus show obviously different in body color, and some geographic populations may present the same difference.

2.	Why do not use high through sequencing technology? The mitochondrial genome of insect contains 37 genes, including 13 protein-encoded genes, 22 tRNA genes, and 2 rRNA genes. So, the other six tRNA genes must present in the mt genome of the parasitoid, just were not identified. And the mt genomes of chalcids exhibit higher recombination ratio, what about the recombination for the parasitoid?

3.	Others need to be corrected:

(1)*Eriosoma lanigerum* (Hausmann) belongs to the order Hemiptera not Hymenoptera；

(2)“Anterior leg“ should be “Fore leg” in Table 2;

(3)“sp.”should be not Italics in Table 4.

……
