# Supplementary material for: Differences in morphology, mitochondrial genomes, and reproductive compatibility between two clades of parasitic wasps Aphelinus mali (Hymenoptera: Aphelindae) in China
Source: PLoS One. 2023 Feb 2;18(2):e0279663. doi: 10.1371/journal.pone.0279663 (PMC9894431; doi:10.1371/journal.pone.0279663)
Supplement: Attachment — Submitted filename: 10-28 rebuttal letter.docx [file pone.0279663.s012.docx]

We changed a funder (31371994) to a new one (32172395).

The funder "National Natural Science Foundation (32172395)" play a role in study design, data collection and preparation of the manuscript, the funder " Taishan Mountain Scholar Constructive Engineering Foundation of Shandong, China " play a role in data collection.

**Reviewer #1:**

The suggestions have been revised.

**Reviewer #6:**

1.The suggestions have been revised

2. About the writing

The English writing had been improved.

**Reviewer #7:**

1. Based on the study of the mitochondrial COI gene of this parasitoid, *A. mali* in China is comprised of two regional clades (named the Shandong and Liaoning clades), we found there were significant difference between two clades of body size, none of body, wing shade and others.

2. Sorry about that, when we did this part of experiment, we chose mitochondrial sequencing first, we will go on the experiment in the future.

3. Others

We have corrected the mistakes.
